# Allele specific expression of Dof genes responding to hormones and abiotic stresses in sugarcane

**DOI:** 10.1371/journal.pone.0227716

**Published:** 2020-01-16

**Authors:** Mingxing Cai, Jishan Lin, Zeyun Li, Zhicong Lin, Yaying Ma, Yibin Wang, Ray Ming

**Affiliations:** 1 College of Life Sciences, Center for Genomics and Biotechnology, Fujian Provincial Key Laboratory of Haixia Applied Plant Systems Biology, Fujian Agriculture and Forestry University, Fuzhou, Fujian, China; 2 College of Crop Sciences, Fujian Agriculture and Forestry University, Fuzhou, Fujian, China; 3 Department of Plant Biology, University of Illinois at Urbana-Champaign, Urbana, IL, United States of America; United Arab Emirates University, UNITED ARAB EMIRATES

## Abstract

Dof transcription factors plant-specific and associates with growth and development in plants. We conducted comprehensive and systematic analyses of Dof transcription factors in sugarcane, and identified 29 *SsDof* transcription factors in sugarcane genome. Those *SsDof* genes were divided into five groups, with similar gene structures and conserved motifs within the same groups. Segmental duplications are predominant in the evolution of *Dof* in sugarcane. *Cis*-element analysis suggested that the functions of *SsDofs* were involved in growth and development, hormones and abiotic stresses responses in sugarcane. Expression patterns indicated that *SsDof7*, *SsDof23* and *SsDof24* had a comparatively high expression in all detected tissues, indicating these genes are crucial in sugarcane growth and development. Moreover, we examined the transcription levels of *SsDofs* under four plant hormone treatments, *SsDof7-3* and *SsDof7-4* were down-regulated after ABA treatment, while *SsDof7-1* and *SsDof7-2* were induced after the same treatment, indicating different alleles may play different roles in response to plant hormones. We also analyzed *SsDofs’* expression profiling under four abiotic stresses, *SsDof5* and *SsDof28* significantly responded to these four stresses, indicating they are associate with abiotic stresses responses. Collectively, our results yielded allele specific expression of *Dof* genes responding to hormones and abiotic stresses in sugarcane, and their *cis*-elements could be crucial for sugarcane improvement.

## Introduction

Dof (DNA-binding with one finger) transcription factors (TFs) are associated with growth and development in plants. A typical DNA-binding domain (C2/C2) exists in all Dof transcription factors and the C2/C2 domain is composed of about 52 amino acids. The C2/C2 domain contains a single zinc finger, which is beneficial for combining the 5 ′-(T/A)AAAG-3 ′ sequence with a conversed target DNA sequence [1]. The C-terminal of Dof transcription factors play important roles in transcription regulation, including interaction with diverse regulatory proteins [2].

The functions of Dof TFs have been identified in many plants. *AtDAG1*, a Dof transcription factor, was identified to be involved in light-quality response in Arabidopsis. In maize, *Dof1* and *Dof2* were identified to promote regulation of carbohydrate metabolism [3]. In potato, researches have been confirmed that Dof transcription factors *StCDF1* was involved in the regulation of tuber development through restraining the expression of *CO1/2* in potato [4]. In rice, over-expression of *OsDof12* promoted early flowering [5]. In tomato, over-expression of Dof transcription factors *SICDF3* promoted late flowering in transgenic Arabidopsis plants [6]. In *Jatropha curcas*, *JcDof3* was regulated by circadian clock and identified to regulate flowering time [7]. Dof transcription factors were also identified to play important roles in plant hormonal signaling. In barley, *HvDof19* was reported to repress the hydrolase gene when the barley aleurone was germinating [8]. *OsDof3* was associate with gibberellin-related expression during germination in rice [9,10]. In Arabidopsis, Dof transcription factor *OBP1* could regulate gene expression when responding to plant hormones, such as salicylic acid and auxin [11]. In addition, previous studies showed that Dof TFs are involved in abiotic stresses responses. In Arabidopsis, *OBP1* was identified to play important roles in regulating the gene expressions responding to the signals of oxidative stresses [11]. In tomato, over-expression of Dof transcription factors *SlCDF1* and *SlCDF3* could influence the salt and drought responses of transgenic plants in Arabidopsis [6].

Dof transcription factors in different plant species have been studied in past years, such as Arabidopsis [12], rice [10], cucumber [13] and soybean [14]. However, information about *Dof* genes is lacking in sugarcane (*Saccharum* spp., Poaceae). Sugarcane is a major crop in producing biofuel and sugar, accounting for about 40% of ethanol production and 80% of sugar production all over the world [15]. The sugarcane (*Saccharum spontaneum*) genome was sequenced and genomic resources are available for detailed analysis of target genes [16]. We performed a comprehensive and systematic analysis to investigate the *Dof* genes in sugarcane genome and 29 *SsDof* genes were identified in sugarcane. These transcription factors were thoroughly analyzed on sequence phylogeny, exon and intron structure, motif patterns, chromosome location, duplication events and *cis*-element analysis. We examined the expression profiling of *SsDofs* in various developmental stages and tissues in sugarcane. We also analyzed the transcription levels of *SsDofs* under different treatments of abiotic stresses and plant hormones.

## Materials and methods

### Plant material and treatments

We used SES208 (*Saccharum spontaneum*, 2n = 8x = 64) as plant materials in our study. And these sugarcane plants grew in the green house at Fujian Agriculture and Forestry University.

For analyzing transcription levels of *SsDof* genes in different tissues and stages: root samples were obtained from root in seedling stage (45 days old), including the top of root (below the root hair, root-t), the middle of root (root-m) and the base of the root (root-b). Stem and leaf samples were from 9 months old premature internode (pre-m-stem3, pre-m-stem6 and pre-m-stem9), 12 months old mature internode (m-stem3, m-stem6 and m-stem9) and leaf (leaf-b, leaf-m and leaf-u) as previously described [17–19].

For analyzing transcription levels of sugarcane *Dof* genes under four plant hormones: the whole sugarcane seedlings (45 days old) were subjected to four plant hormones (ABA, GA, Auxin and Ethylene, purchased from Solarbio company), the leaves were collected at 24 h after treatments.

For analyzing transcription levels of sugarcane *Dof* genes in seedling stage and under four treatments by RT-qPCR: tissue samples were obtained from root, stem and leaf in seedling stage (45 days old). As for the cold and heat applications, the sugarcane seedlings were grown at 4°C and 38°C (artificial climate chamber from Yiheng company) for 4, 8, 12 and 24 h, respectively. In addition, the whole seedlings were performed with 15% PEG6000 (purchased from Takara company) and 100 mM NaCl (purchased from Takara company) for 4, 8, 12 and 24 h respectively.

### Identification of *Dof* genes in Saccharum spontaneum

We obtained the sequences of *Dof* genes in *Arabidopsis thaliana* and *Oryza sativa* from Arabidopsis genome (http://www.arabidopsis.org/) and rice genome (http://rice.plantbiology.msu.edu/). Then we performed BLASTN to identify all Dof homolog hits in *Saccharum spontaneum* genome. We collected all non-redundant hits whose values were less than 1E^-5^. And we used the PFAM program (http://pfam.sanger.ac.uk/) and SMART program (http://smart.embl-heidelberg.de/) to further confirm the existence of *Dof* domain (PF002701).Then we used the GENSCAN program (http://genes.mit.edu/GENSCAN.html) to verify the sequences identified [20]. We used the ExPASy program (https://web.expasy.org/protparam/) to check the molecular weights (MW) and isoelectric points (PI) of all sequences.

### Sequence analysis

We performed the ClustalW to investigate multiple sequence alignments of *SsDof* protein sequences. We checked the distribution of amino-acids of *SsDof* domains with WebLogo program (http://weblogo.berkeley.edu/logo.cgi). By performing GSDS program (http://gsds.cbi.pku.edu.cn) [21], we investigated exon and intron compositions of *SsDof* genes. We checked conserved motifs composition of sugarcane *Dof* proteins by MEME program (http://meme.nbcr.net/meme/intro.html) [22].

### Phylogenetic analysis of *SsDof* genes in sugarcane

Based on multiple sequence alignments of *SsDof* and *AtDof* proteins and all sugarcane *Dof* genes could be divided into various groups. We performed phylogenetic analysis with MEGA5.0. Sequence of *Dof* proteins from Arabidopsis and sorghum were obtained from literature [23]. The phylogenetic tree image was enhanced by the Evolview online program (http://www.evolgenius.info/evolview).

### Chromosomal distribution and gene duplication

The genomic and CDS sequences of *SsDof* genes were obtained from *Saccharum spontaneum* genome. We checked the gene duplications of *SsDof* genes by BLAST search in the genome. The chromosomal distribution of *SsDof* genes was generated by Circos software (http://circos.ca/).

### Ka/Ks values of the sugarcane *Dof* genes

We investigated the nonsynonymous substitution rate (Ka) and synonymous substitution rate (Ks) with KaKs_Calculator v2.0 [24,25]. We calculated the divergence time of *SsDof* genes with the formula T = Ks/ (2 ×6.1 ×10^−9^) ×10^−6^ Mya [26].

### *Cis*-element analysis

We extracted the 1.5kb upstream sequence of *SsDof* genes promoter. With the PlantPAN program [27] (http://plantpan.itps.ncku.edu.tw/) and PlantCARE program [28] (http://bioinformatics.psb.ugent.be/webtools/plantcare/html/), we investigated the *cis*-elements of *SsDof* genes and collected the *cis*-element about growth and development, abiotic stresses and hormones responses in plant. The heatmap of *cis*-elements of *SsDof* genes was performed by TBtools software [29].

### Expression profiling of sugarcane *Dof* genes by RNA-seq

RNA-seq was carried out using Illumina NovaSeq. We use the *S*. *spontaneum* AP85-441 genome as the reference genome to align the reads of SES208. Using Trinity software (https://github.com/trinityrnaseq/trinityrnaseq/wiki), we counted mappable reads from SES208 and normalized the FPKM values of each sample. Then, we used the TBtools software to generate the heatmap. RPKM value of *SsDofs* used in this study were shown in S5 Table.

### Expression levels of *SsDof* genes based on qRT-PCR

We isolated RNA of sugarcane sample using Trizol [30] (purchased from Solarbio company). The Roche Lightcyler® 480 instrument was used to perform the quantitative RT-PCR. We selected the *GAPDH* (glyceraldehyde-3-phosphate dehydrogenase) gene as the internal standards for normalization [31], and each treatment was carried out with three replications. The expression levels of *SsDof* genes were calculated by the 2^-ΔΔCt^ methods [32]. The primers of *SsDofs* performed were shown in S6 Table.

## Results

### Identification of *SsDof* genes in sugarcane

29 *SsDof* genes were identified in the sugarcane *S*. *spontaneum* AP85-441 genome and these *SsDof* genes were named as *SsDof1*-*SsDof29*. The alleles, tandem duplicates and paralogs of each *SsDof* are named by “-1” to “-7” with gene name (Table 1). Among these 29 *SsDofs*, four *SsDofs* have four alleles (*SsDof5*, *SsDof6*, *SsDof7* and *SsDof13*), ten *SsDofs* have three alleles (*SsDof1*, *SsDof3*, *SsDof11*, *SsDof12*, *SsDof20*, *SsDof22*, *SsDof25*, *SsDof26*, *SsDof27* and *SsDof28*), ten *SsDofs* have two alleles (*SsDof2*, *SsDof4*, *SsDof9*, *SsDof14*, *SsDof15*, *SsDof16*, *SsDof18*, *SsDof19*, *SsDof23* and *SsDof24*), five *SsDofs* have only one alleles (*SsDof8*, *SsDof10*, *SsDof17*, *SsDof21*, *SsDof29*). In addition, ten *SsDofs* have one paralog (*SsDof4*, *SsDof6*, *SsDof18*, *SsDof20*, *SsDof22*, *SsDof24*, *SsDof25*, *SsDof26*, *SsDof27* and *SsDof28*), *SsDof8* and *SsDof13* have two paralogs, *SsDof1* have four paralogs. *SsDof1*, *SsDof8* and *SsDof20* have one tandem duplicate respectively (S1 Table).

**Table 1:** Identification of the alleles and duplicates of *SsDof* genes in sugarcane.

Gene Name	Allele-A	Allele-B	Allele-C	Allele-D	Tandem Duplicate	Paralogous
*SsDof1*	*SsDof1-1*	*SsDof1-4*	*-*	*SsDof1-5*	*SsDof1-2*	*SsDof1-3*
						*SsDof1-6*
						*SsDof1-7*
*SsDof2*	*SsDof2-1*	*SsDof2-2*	*-*	*-*	*-*	*-*
*SsDof3*	*SsDof3-1*	*-*	*SsDof3-2*	*SsDof3-3*	*-*	*-*
*SsDof4*	*-*	*SsDof4-1*	*-*	*SsDof4-3*	*-*	*SsDof4-2*
*SsDof5*	*SsDof5-1*	*SsDof5-2*	*SsDof5-3*	*SsDof5-4*	*-*	*-*
*SsDof6*	*SsDof6-1*	*SsDof6-2*	*SsDof6-3*	*SsDof6-4*	*-*	*SsDof6-5*
*SsDof7*	*SsDof7-1*	*SsDof7-2*	*SsDof7-3*	*SsDof7-4*	*-*	*-*
*SsDof8*	*-*	*-*	*SsDof8-1*	*-*	*SsDof8-3*	*SsDof8-2*
*SsDof9*	*SsDof9-1*	*-*	*SsDof9-2*	*-*	*-*	*-*
*SsDof10*	*SsDof10*	*-*	*-*	*-*	*-*	*-*
*SsDof11*	*-*	*SsDof11-1*	*SsDof11-2*	*SsDof11-3*	*-*	*-*
*SsDof12*	*SsDof12-1*	*SsDof12-2*	*SsDof12-3*	*-*	*-*	*-*
*SsDof13*	*SsDof13-1*	*SsDof13-2*	*SsDof13-3*	*SsDof13-5*	*-*	*SsDof13-4*
						*SsDof13-6*
*SsDof14*	*-*	*SsDof14-1*	*SsDof14-2*	*-*	*-*	*-*
*SsDof15*	*-*	*SsDof15-1*	*-*	*SsDof15-2*	*-*	*-*
*SsDof16*	*-*	*-*	*SsDof16-1*	*SsDof16-2*	*-*	*-*
*SsDof17*	*-*	*-*	*SsDof17*	*-*	*-*	*-*
*SsDof18*	*SsDof18-1*	*-*	*SsDof18-2*	*-*	*-*	*SsDof18-3*
*SsDof19*	*-*	*SsDof19-1*	*SsDof19-2*	*-*	*-*	*-*
*SsDof20*	*-*	*SsDof20-1*	*SsDof20-2*	*SsDof20-4*	*SsDof20-3*	*-*
*SsDof21*	*-*	*-*	*SsDof21*	*-*	*-*	*-*
*SsDof22*	*-*	*SsDof22-1*	*SsDof22-2*	*SsDof22-3*	*-*	*SsDof22-4*
*SsDof23*	*SsDof23-1*	*-*	*-*	*SsDof23-2*	*-*	*-*
*SsDof24*	*SsDof24-1*	*SsDof24-3*	*-*	*-*	*-*	*SsDof24-2*
*SsDof25*	*-*	*SsDof25-1*	*SsDof25-3*	*SsDof25-4*	*-*	*SsDof25-2*
*SsDof26*	*SsDof26-2*	*-*	*SsDof26-3*	*SsDof26-4*	*-*	*SsDof26-1*
*SsDof27*	*SsDof27-1*	*SsDof27-3*	*-*	*SsDof27-4*	*-*	*SsDof27-2*
*SsDof28*	*SsDof28-1*	*SsDof28-2*	*SsDof28-4*	*-*	*-*	*SsDof28-3*
*SsDof29*	*SsDof29*	*-*	*-*	*-*	*-*	*-*

The Open Reading Frame length of *SsDofs* ranged from 504 bp (*SsDof15-2*) to 2337 bp (*SsDof13-4*) (Tables 2 and 3). The encoding peptides of *SsDofs* ranged 167 to 778 amino acids. The molecular weight (Mw) of *SsDofs* ranged from 17096.27 Da to 86535.27 Da. The theoretical PI values of *SsDofs* varied from 4.74 (*SsDof17*) to 11.58 (*SsDof26-2*).

**Table 2:** Characterization of *Dof* genes in *Saccharum spontaneum*.

Gene name	Gene ID	Gene location	ORF length (bp)	Amino Acids	MW(Da)	PI
*SsDof1-1*	*Sspon*.*001A0039820*	Chr1A: 107277015–107278072	1032	343	33855.37	9.34
*SsDof1-2*	*Sspon*.*001A0040020*	Chr1A: 107568808–107570208	1095	364	36620.34	9.84
*SsDof1-3*	*Sspon*.*001A0040040*	Chr1A: 107588205–107589608	1188	395	38827.05	9.72
*SsDof1-4*	*Sspon*.*001B0041380*	Chr1B: 107251810–107253383	1269	422	42103.38	9.34
*SsDof1-5*	*Sspon*.*001D0049380*	Chr1D: 116301199–116302320	1122	374	37009.86	9.59
*SsDof1-6*	*Sspon*.*003A0031600*	Chr3A: 76875402–76876478	1077	359	35604.47	9.59
*SsDof1-7*	*Sspon*.*007C0001360*	Chr7C: 3129542–3130585	1044	347	34246.8	9.59
*SsDof2-1*	*Sspon*.*001A0036180*	Chr1A: 100341035–100342153	1119	372	37928.83	9.33
*SsDof2-2*	*Sspon*.*001B0043630*	Chr1B: 111139532–111140650	1119	372	37896.72	9.33
*SsDof3-1*	*Sspon*.*001A0029370*	Chr1A: 84716423–84717322	900	300	30706.21	9.19
*SsDof3-2*	*Sspon*.*001C0028070*	Chr1C: 83054119–83054961	843	281	29040.4	9.19
*SsDof3-3*	*Sspon*.*001D0029560*	Chr1D: 82134084–82135031	948	315	32307.15	9.02
*SsDof4-1*	*Sspon*.*001B0032400*	Chr1B: 86475732–86476847	1116	371	36959.1	8.76
*SsDof4-2*	*Sspon*.*001B0046520*	Chr1B: 121056996–121058138	1143	380	38041.32	8.42
*SsDof4-3*	*Sspon*.*001D0028210*	Chr1D: 79915954–79917051	1098	366	36338.32	8.76
*SsDof5-1*	*Sspon*.*001A0025480*	Chr1A: 76225817–76229102	1953	650	69432.29	9.79
*SsDof5-2*	*Sspon*.*001B0029870*	Chr1B: 81048181–81049070	789	262	26704.79	9.44
*SsDof5-3*	*Sspon*.*001C0025912*	Chr1C: 78126780–78128026	777	258	26196.25	9.44
*SsDof5-4*	*Sspon*.*001D0026050*	Chr1D: 73926730–73928271	1152	383	40006.11	10.18
*SsDof6-1*	*Sspon*.*001A0021890*	Chr1A: 65430766–65432971	1377	458	48243.38	6.43
*SsDof6-2*	*Sspon*.*001B0025890*	Chr1B: 69119245–69122095	1374	457	48203.36	6.53
*SsDof6-3*	*Sspon*.*001C0022160*	Chr1C: 67425393–67427635	1377	458	48445.64	6.75
*SsDof6-4*	*Sspon*.*001D0022120*	Chr1D: 62210443–62212843	1383	460	48565.98	7.18
*SsDof6-5*	*Sspon*.*008B0007501*	Chr8B: 14418415–14421292	1383	460	48643.91	6.76
*SsDof7-1*	*Sspon*.*001A0004310*	Chr1A: 11011237–11013835	1275	424	45154.75	8.65
*SsDof7-2*	*Sspon*.*001B0004180*	Chr1B: 9479789–9482098	1245	415	44229.74	8.55
*SsDof7-3*	*Sspon*.*001C0004410*	Chr1C: 10339700–10342509	1275	424	45168.77	8.65
*SsDof7-4*	*Sspon*.*001D0003951*	Chr1D: 9338479–9341224	1275	424	45168.77	8.65
*SsDof8-1*	*Sspon*.*001C0007723*	Chr1C: 17803173–17805227	1167	388	39864.4	10.03
*SsDof8-2*	*Sspon*.*001C0007730*	Chr1C: 17821184–17821912	1160	387	39850.5	10.01
*SsDof8-3*	*Sspon*.*001C0008341*	Chr1C: 19056918–19058977	1170	389	40063.61	10.12
*SsDof9-1*	*Sspon*.*002A0015730*	Chr2A: 32407233–32408012	780	259	26568.5	9.04
*SsDof9-2*	*Sspon*.*002C0016840*	Chr2C: 36552182–36552967	786	261	26826.73	8.72
*SsDof10*	*Sspon*.*002A0009300*	Chr2A: 20582673–20583731	1059	352	34789.78	8.15
*SsDof11-1*	*Sspon*.*002B0000581*	Chr2B: 3632543–3635779	1476	491	52335.49	7.01
*SsDof11-2*	*Sspon*.*002C0001090*	Chr2C: 3277887–3280693	1383	460	48722.43	7.45
*SsDof11-3*	*Sspon*.*002D0001620*	Chr2D: 4031370–4038967	1335	445	47424.38	8.46
*SsDof12-1*	*Sspon*.*003A0028620*	Chr3A: 69989363–69990058	696	231	23501.82	9.76
*SsDof12-2*	*Sspon*.*003B0032940*	Chr3B: 92445084–92445779	696	231	23545.88	9.76
*SsDof12-3*	*Sspon*.*003C0036300*	Chr3C: 88678264–88678968	705	235	23979.32	9.9
*SsDof13-1*	*Sspon*.*003A0006040*	Chr3A: 13413940–13417033	1680	560	61340.56	5.07
*SsDof13-2*	*Sspon*.*003B0026381*	Chr3B: 77706045–77710103	1686	561	61372.44	5.19
*SsDof13-3*	*Sspon*.*003C0029740*	Chr3C: 74892972–74896704	1644	548	60012.04	5.34
*SsDof13-4*	*Sspon*.*003C0029780*	Chr3C: 74907257–74917001	1630	530	60007.37	5.29

**Table 3:** Characterization of *Dof* genes in *Saccharum spontaneum*.

Gene name	Gene ID	Gene location	ORF length (bp)	Amino Acids	MW(Da)	pI
*SsDof13-5*	*Sspon*.*003D0018600*	Chr3D: 40904961–40906067	1560	520	56950.86	5.36
*SsDof13-6*	*Sspon*.*003D0018660*	Chr3D: 40995672–40999792	1686	561	61258.33	5.19
*SsDof14-1*	*Sspon*.*003B0025800*	Chr3B: 76440052–76443429	1473	490	52633.24	9.09
*SsDof14-2*	*Sspon*.*003C0028740*	Chr3C: 72777112–72781127	1476	491	52782.25	8.81
*SsDof15-1*	*Sspon*.*003B0014700*	Chr3B: 32136655–32137455	528	176	18244.55	9.59
*SsDof15-2*	*Sspon*.*003D0013530*	Chr3D: 30080206–30080709	504	167	17096.27	10.06
*SsDof16-1*	*Sspon*.*003C0015340*	Chr3C: 32439780–32440484	705	234	25168	9.34
*SsDof16-2*	*Sspon*.*003D0009930*	Chr3D: 21511270–21511971	702	233	25024.9	9.34
*SsDof17*	*Sspon*.*003C0009580*	Chr3C: 20349691–20350686	996	331	34765.62	4.74
*SsDof18-1*	*Sspon*.*004A0005590*	Chr4A: 13321550–13322452	903	300	30570.02	8.96
*SsDof18-2*	*Sspon*.*004C0005730*	Chr4C: 13366465–13367376	912	303	30917.39	8.81
*SsDof18-3*	*Sspon*.*001D0049210*	Chr1D: 115935252–115936160	909	302	30820.27	8.81
*SsDof19-1*	*Sspon*.*004B0005410*	Chr4B: 11460898–11463432	885	294	30771.02	8.51
*SsDof19-2*	*Sspon*.*004C0003870*	Chr4C: 9130210–9133430	894	297	31130.46	8.32
*SsDof20-1*	*Sspon*.*004B0006870*	Chr4B: 14469135–14471470	1344	448	44998.9	9.19
*SsDof20-2*	*Sspon*.*004C0007880*	Chr4C: 17749498–17751838	1335	444	44862.7	8.6
*SsDof20-3*	*Sspon*.*004C0007890*	Chr4C: 17761529–17762605	1077	359	36120.92	8.97
*SsDof20-4*	*Sspon*.*004D0009220*	Chr4D: 19193994–19196498	1347	448	45289.23	9.28
*SsDof21*	*Sspon*.*007C0023290*	Chr7C: 73009957–73013461	1080	359	38621.19	9.27
*SsDof22-1*	*Sspon*.*005B0004970*	Chr5B: 10549765–10550844	1080	359	35704.3	8.67
*SsDof22-2*	*Sspon*.*005C0003500*	Chr5C: 8881598–8884191	1260	419	42896.68	9.37
*SsDof22-3*	*Sspon*.*005D0008750*	Chr5D: 18173443–18174492	1050	350	34939.46	8.67
*SsDof22-4*	*Sspon*.*006B0011060*	Chr6B: 35181618–35182691	1074	357	35608.21	8.67
*SsDof23-1*	*Sspon*.*005A0001180*	Chr5A: 2756149–2757488	657	218	22897.28	6.4
*SsDof23-2*	*Sspon*.*005D0000820*	Chr5D: 2072384–2073390	663	220	22923.32	6.94
*SsDof24-1*	*Sspon*.*006A0001010*	Chr6A: 2592979–2593749	771	256	26513.39	6.52
*SsDof24-2*	*Sspon*.*006A0001071*	Chr6A: 2715849–2716619	771	256	26518.4	6.43
*SsDof24-3*	*Sspon*.*006B0000220*	Chr6B: 1068674–1069444	771	256	26465.34	5.97
*SsDof25-1*	*Sspon*.*002B0036670*	Chr2B: 103776333–103780462	1116	371	37965.37	9.16
*SsDof25-2*	*Sspon*.*002B0036710*	Chr2B: 103836417–103840761	1077	358	36590.8	9.12
*SsDof25-3*	*Sspon*.*002C0041860*	Chr2C: 114639384–114641320	1026	342	34622.62	9.03
*SsDof25-4*	*Sspon*.*002D0036520*	Chr2D: 99387218–99388215	957	318	32106.81	9.34
*SsDof26-1*	*Sspon*.*001D0041800*	Chr1D: 104783322–104784158	837	278	28185.29	9.17
*SsDof26-2*	*Sspon*.*002A0040110*	Chr2A: 108980068–108980730	663	220	22534.49	11.58
*SsDof26-3*	*Sspon*.*002C0040670*	Chr2C: 111144346–111145161	816	271	27706.73	9.54
*SsDof26-4*	*Sspon*.*002D0035330*	Chr2D: 96117145–96117990	846	281	28481.63	9.54
*SsDof27-1*	*Sspon*.*007A0015970*	Chr7A: 43896449–43897984	1110	365	37898.48	9.73
*SsDof27-2*	*Sspon*.*007A0015980*	Chr7A: 43901631–43904131	1083	360	37119.77	9.59
*SsDof27-3*	*Sspon*.*007B0020182*	Chr7B: 59717402–59719469	1131	376	38340.61	9.18
*SsDof27-4*	*Sspon*.*007D0018410*	Chr7D: 53199255–53201078	1116	371	37977.3	9.2
*SsDof28-1*	*Sspon*.*007A0005700*	Chr7A: 11061126–11061755	630	210	22185.16	9.85
*SsDof28-2*	*Sspon*.*007B0005260*	Chr7B: 10048038–10053223	642	213	22896.8	10.16
*SsDof28-3*	*Sspon*.*007B0005270*	Chr7B: 10056998–10062050	579	192	21126.03	11.41
*SsDof28-4*	*Sspon*.*007C0003290*	Chr7C: 6795289–6795957	669	222	23309.36	9.99
*SsDof29*	*Sspon*.*007A0009260*	Chr7A: 18472446–18473539	1020	339	35916.31	5.44

**Fig 1:**
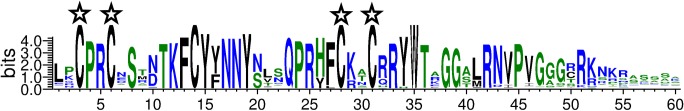
The distribution of all sugarcane Dof domains at each position. Multiple alignments of all sugarcane Dof domains were performed to plot the sequence logos with ClustalW. The information content of Dof domains at each position was indicated with the bits score. The four conserved cysteine residues of SsDof domains were indicated with the asterisks.

To explore the distribution of the homologous sequences at each position, we performed the multiple alignment analysis with *SsDofs*’ amino acid sequences. It was indicated that all *SsDofs* possess a representative DNA binding domain of 52 amino acids that included a single C2/C2 zinc finger structure. (Fig 1).

### Phylogenetic relationships of *Dof* genes in sugarcane, sorghum and Arabidopsis

**Fig 2:**
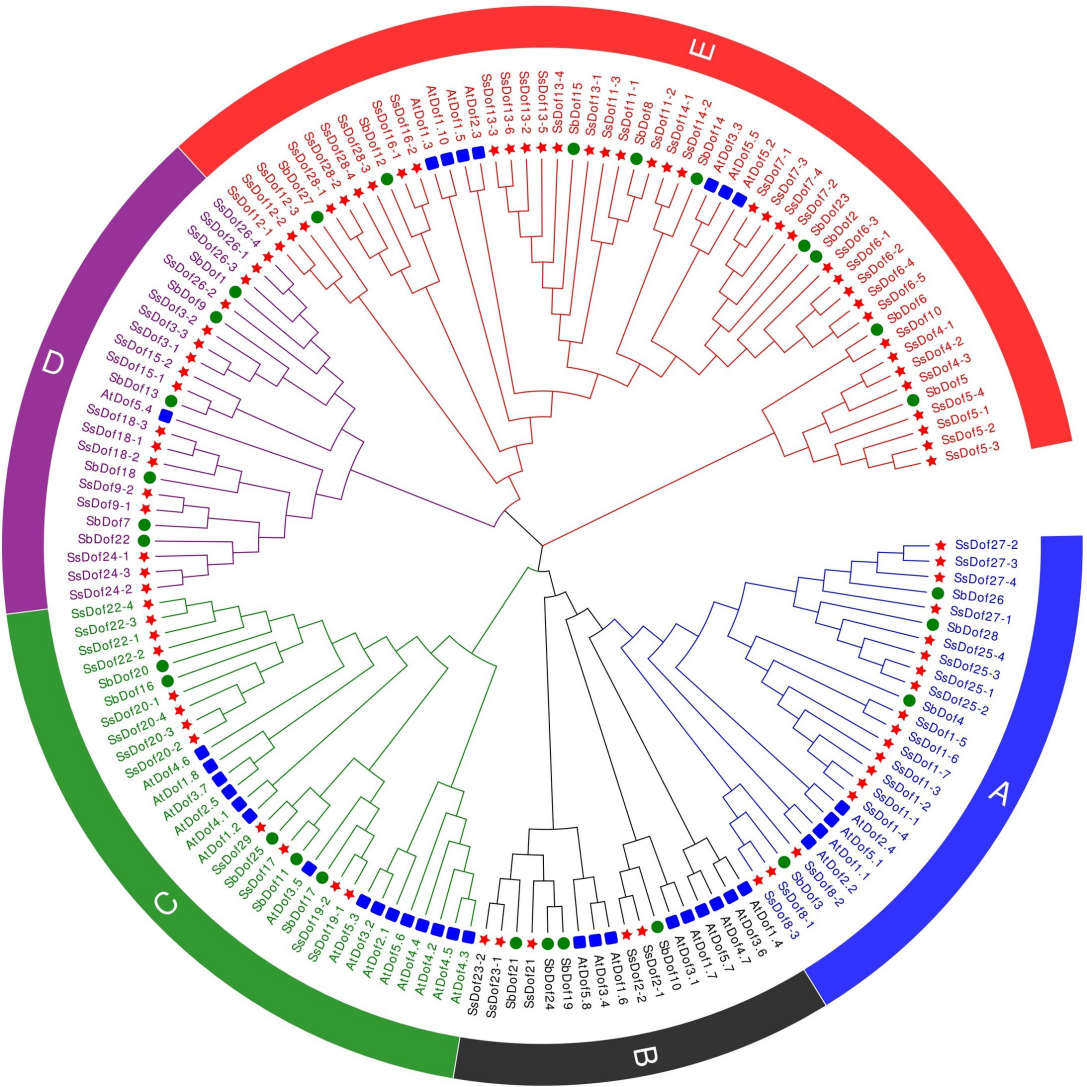
Phylogenetic relationships of *Dof* genes in sugarcane, sorghum and Arabidopsis. All *Dof* genes were divided into five groups (A, B, C, D and E) with different color arcs. The blue squares, green circles and red asterisks represent *Dof* genes from Arabidopsis, sorghum, and sugarcane, respectively.

The amino acid sequences of all *SsDofs* with 36 *AtDofs* [33] and 28 *SbDofs* [34] were used to construct an unrooted phylogenetic tree (Fig 2 and S2 Table). Similar to earlier reports of *AtDofs*, the Dof proteins of three plants would be divided to five groups (group A, B, C, D and E). Group E contains the most *Dof* genes (53), accounting for 34.6%. Group A, B, C and D contain 26, 18, 32 and 24 *Dof* genes, respectively. Additionally, five *SsDof* genes belong to Group A (*SsDof1*, *SsDof4*, *SsDof8*, *SsDof25*, *SsDof27*); three *SsDof* genes belong to Group B (*SsDof2*, *SsDof21*, *SsDof23*); five *SsDof* genes belong to Group C (*SsDof17*, *SsDof19*, *SsDof20*, *SsDof22*, *SsDof29*); six *SsDof* genes belong to Group C (*SsDof3*, *SsDof9*, *SsDof15*, *SsDof18*, *SsDof24*, *SsDof26*); eleven *SsDof* genes belong to Group E (*SsDof4*, *SsDof5*, *SsDof6*, *SsDof7*, *SsDof10*, *SsDof11*, *SsDof12*, *SsDof13*, *SsDof14*, *SsDof16* and *SsDof28*). Based on the phylogenetic tree, five pairs of putative orthologs from *Saccharum spontaneum* and *Sorghum bicolor* were also identified, such as *SsDof29*/*SbDof25*, *SsDof17*/*SbDof11*, *SsDof15-1*/*SbDof13*, *SsDof11-2*/*SbDof8* and *SsDof10*/*SbDof6*.

### Motif composition and gene structure of sugarcane *Dof* gene family

**Fig 3:**
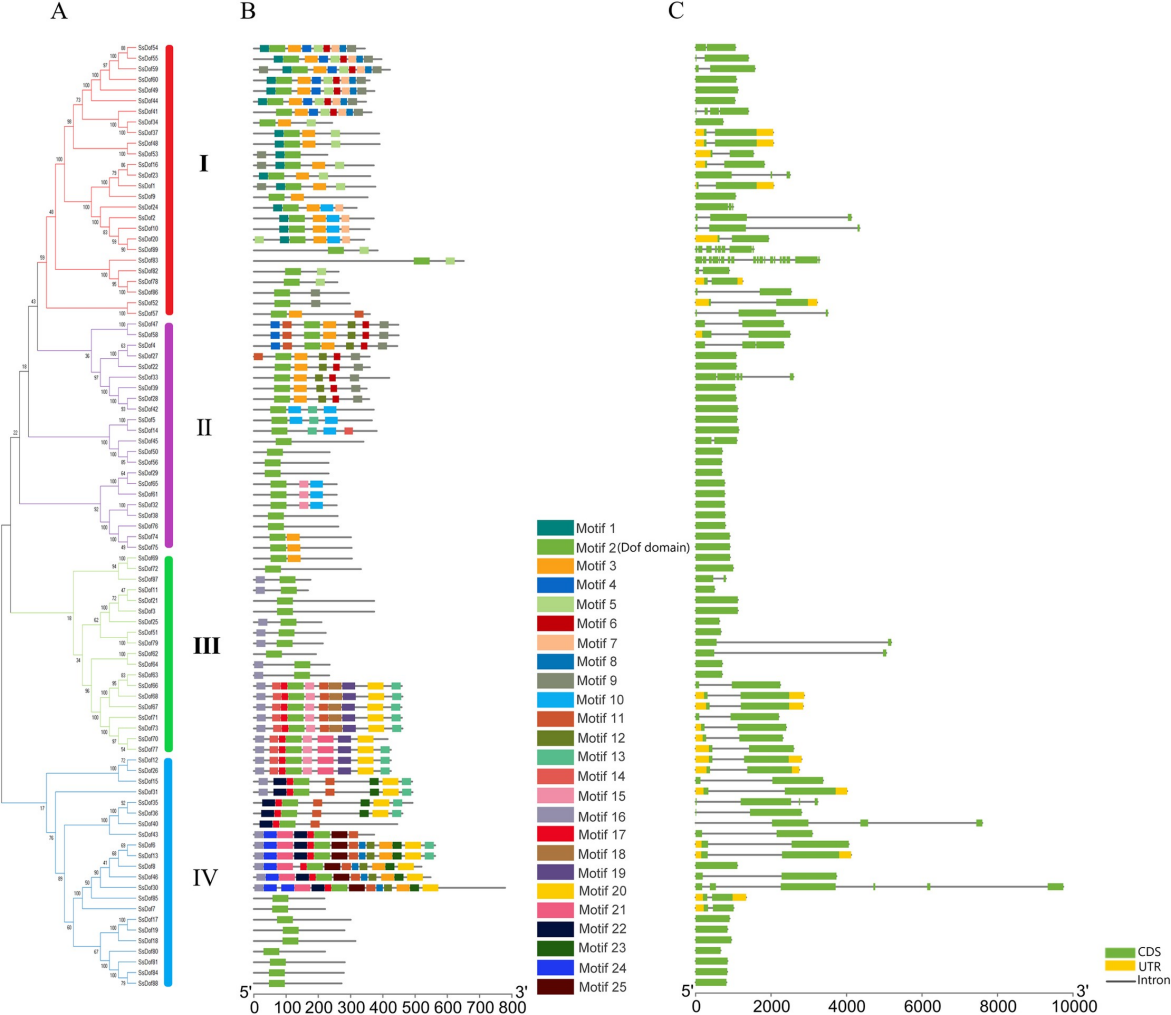
Phylogenetic relationship, conserved protein motifs and gene structures of *SsDof* genes in sugarcane. (A) The phylogenetic tree was based on multiple sequence alignments of SsDof proteins using MEGA 5 and divided into four groups (Ⅰ, Ⅱ, Ⅲ and Ⅳ). (B) The conserved motif of SsDof proteins. The motifs are shown in different color boxes with numbers 1–25. (C) The gene structures of *SsDof* genes. The green boxes indicate CDS; the yellow boxes indicate untranslated 5′- and 3′-regions; the black lines indicate introns.

We performed the MEME program to investigate the motif patterns of SsDof proteins. And 25 motifs were checked in SsDofs protein sequences (Fig 3A and 3B). Similar to the results in Arabidopsis [35], soybean [14], cucumber [13] and tomato [36], our results suggested that *SsDof* genes were highly conserved in sugarcane. The motif1 was the conserved Dof domain and distributed in each SsDof proteins. In addition, the motif patterns of SsDof proteins have similar compositions within the same group. For instance, in group Ⅰ, 10 motifs (1, 4, 7, 8, 10, 11, 15, 16, 21, 25) were the conserved motifs. There were 12 conserved motifs (1, 7, 8, 11, 12, 13, 14, 15, 16, 18, 21, 24) in group Ⅱ. And group Ⅲ contained the most numbers of motifs, including 17 conserved motifs, while group Ⅳ had only one conserved motif (Dof domain). These results indicated that there would be some similar functions of *SsDof* genes within the same group.

To investigate the evolution of *SsDof* genes in sugarcane, we examined the gene structure of *SsDof* genes. As depicted in Fig 3A and 3C, the number of introns of *SsDofs* was no more than 5. Thirty-nine (43.8%) alleles and paralogs were intronless, whereas thirty-seven (41.6%) alleles and paralogs contained one intron. In addition, some *SsDofs* groups showed similar gene structure compositions. For instance, *SsDofs* in group Ⅲ had the most numbers of introns, in which *SsDof13-4* had five introns. *SsDofs* in group Ⅳ were intronless except *SsDof23* including one intron. In groupⅠ, the number of introns of *SsDofs* various from 0 to 3.

### Chromosomal location and duplication of sugarcane *Dof* genes

**Fig 4:**
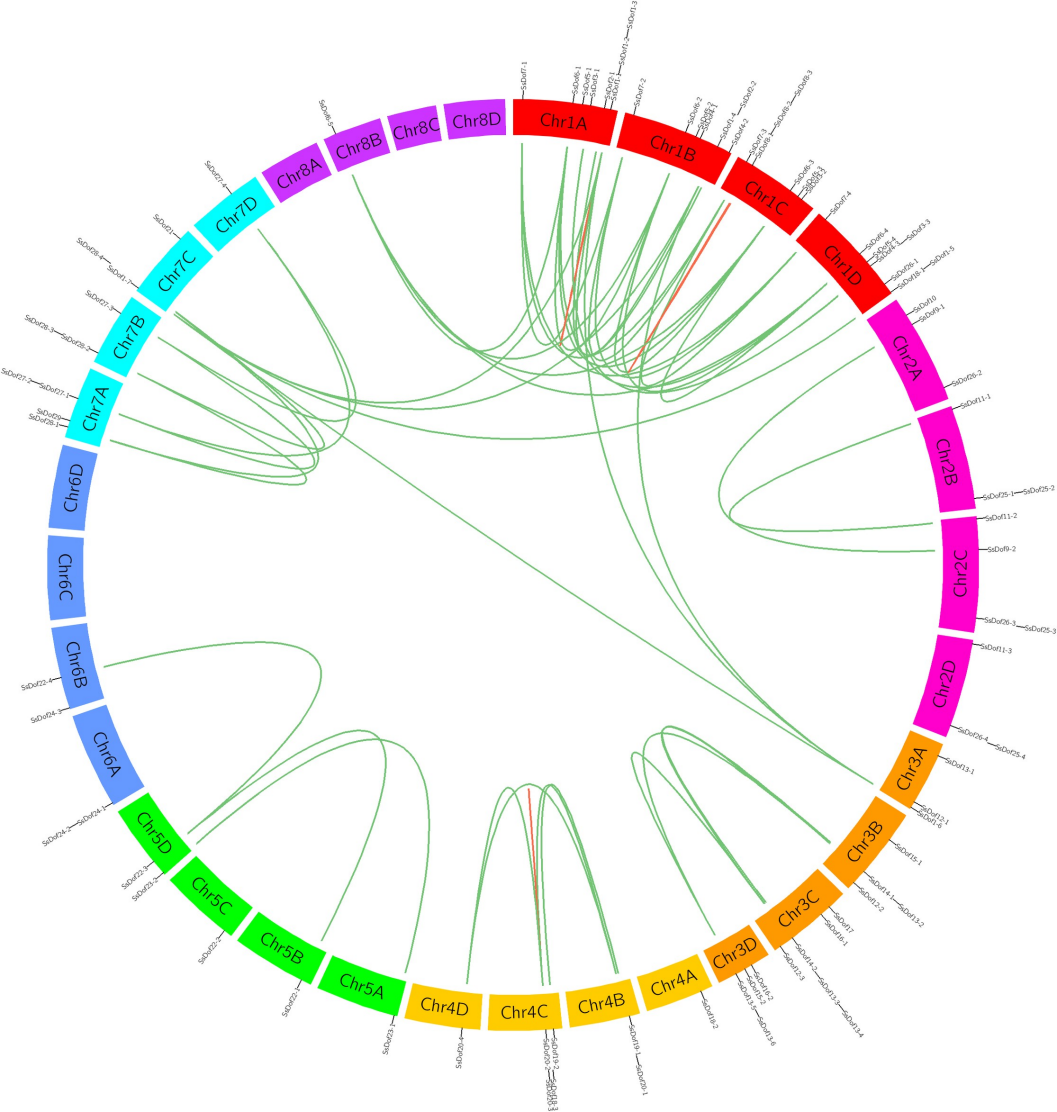
Chromosomal locations of the *SsDof* genes in *S*.*spontaneum*. Green lines indicate segmental duplication gene pairs, while the red lines show tandem duplication gene pairs. The chromosome numbers are shown in the center of sugarcane chromosomes.

*SsDofs* were unevenly distributed in 27 of the 32 chromosomes of *S*. *spontaneum* AP85-441 except chromosome 6C, 6D, 8A, 8C and 8D (Fig 4). Chromosome 1A and 1D contained eight *SsDofs* followed by seven *SsDofs* in chromosomes 1B and 1C. There was only one *SsDofs* in chromosomes 4A, 4D, 5A, 5B, 5C, 7D and 8B. There was no correlation between the number of *SsDof* genes and the length of sugarcane chromosomes.

Transposition events, tandem and segmental duplications are the primary reasons of gene family expansions [37]. Tandem duplication events happen when two or more genes duplicate within 200kb chromosome region [38], while segmental duplication events mean gene duplications happened in different chromosomes [39]. In this study, 49 pairs of duplicated genes were identified (S3 Table). Among these duplicated *SsDof* gene pairs, three gene pairs are tandem duplications (*SsDof1-1*/*SsDof1-2*, *SsDof8-1*/*SsDof8-3*, *SsDof20-2*/*SsDof20-3*), and the other forty-six gene pairs belong to segmental duplications.

The Ks, Ka and Ka/Ks ratio were calculated to investigate the divergence time of the duplication blocks. The duplications of *SsDofs* in *S*. *spontaneum* AP85-441 occurred approximately 0.21 Mya (million years ago) to 15.60 Mya with an average of 1.97 Mya (Table 4). *SsDof1*, *SsDof2*, *SsDof3*, *SsDof11*, *SsDof14*, *SsDof20*, *SsDof23*, *SsDof27* and *SsDof28* had undergone purifying selection because their Ka/Ks ratio were lower than 1, whereas *SsDof8* had undergone positive selection as its Ka/Ks ratio was higher than 1. These results indicate that different *SsDofs* were under different selective constraints relating to their functions.

**Table 4:** Duplicated *SsDof* genes and the divergence time of *SsDof* genes.

Gene1	Gene2	Duplication Type	Ka	Ks	Ka/Ks	Divergence time (Mya)
*SsDof1-1*	*SsDof1-2*	Tandem duplication	0.0594	0.0941	0.6313	7.72
*SsDof1-3*	*SsDof1-4*	Segmental duplication	0.0149	0.023	0.6493	1.88
*SsDof1-3*	*SsDof1-6*	Segmental duplication	0.0013	0.0178	0.0714	1.46
*SsDof1-3*	*SsDof1-7*	Segmental duplication	0.004	0.0182	0.2169	1.49
*SsDof1-4*	*SsDof1-6*	Segmental duplication	0.0025	0.0178	0.1427	1.46
*SsDof1-4*	*SsDof1-7*	Segmental duplication	0.0053	0.0183	0.2888	1.5
*SsDof1-5*	*SsDof1-7*	Segmental duplication	0.0013	0.011	0.1204	0.9
*SsDof1-6*	*SsDof1-7*	Segmental duplication	0.0027	0.0038	0.725	0.31
*SsDof2-1*	*SsDof2-2*	Segmental duplication	0.0037	0.0134	0.2754	1.1
*SsDof3-3*	*SsDof3-1*	Segmental duplication	0.0148	0.0505	0.2923	4.14
*SsDof6-1*	*SsDof6-2*	Segmental duplication	0.0029	0.0031	0.9474	0.25
*SsDof6-1*	*SsDof6-3*	Segmental duplication	0.0048	0.0061	0.7889	0.5
*SsDof6-1*	*SsDof6-4*	Segmental duplication	0.0126	0.0216	0.5827	1.77
*SsDof6-1*	*SsDof6-5*	Segmental duplication	0.0058	0.0031	1.8997	0.25
*SsDof6-2*	*SsDof6-3*	Segmental duplication	0.0019	0.0031	0.6332	0.25
*SsDof6-2*	*SsDof6-4*	Segmental duplication	0.0097	0.0185	0.5238	1.52
*SsDof6-2*	*SsDof6-5*	Segmental duplication	0.0029	0.0061	0.4745	0.5
*SsDof6-3*	*SsDof6-4*	Segmental duplication	0.0087	0.0215	0.4035	1.77
*SsDof6-3*	*SsDof6-5*	Segmental duplication	0.0019	0.003	0.6323	0.25
*SsDof6-4*	*SsDof6-5*	Segmental duplication	0.0106	0.0245	0.4325	2.01
*SsDof7-1*	*SsDof7-2*	Segmental duplication	0.0074	0.0068	1.08	0.56
*SsDof7-1*	*SsDof7-4*	Segmental duplication	0.001	0.0033	0.3099	0.27
*SsDof7-2*	*SsDof7-3*	Segmental duplication	0.0063	0.0068	0.9251	0.56
*SsDof7-2*	*SsDof7-4*	Segmental duplication	0.0063	0.0103	0.6153	0.84
*SsDof8-1*	*SsDof8-3*	Tandem duplication	0.0047	0.0033	1.4225	0.27
*SsDof11-1*	*SsDof11-2*	Segmental duplication	0.0362	0.101	0.3583	8.28
*SsDof13-2*	*SsDof13-3*	Segmental duplication	0.004	0.0054	0.7339	0.45
*SsDof13-2*	*SsDof13-6*	Segmental duplication	0.0031	0.0026	1.1799	0.21
*SsDof13-3*	*SsDof13-6*	Segmental duplication	0.0008	0.0082	0.0977	0.67
*SsDof14-1*	*SsDof14-2*	Segmental duplication	0.0054	0.0175	0.3059	1.44
*SsDof20-1*	*SsDof20-2*	Segmental duplication	0.0143	0.024	0.5981	1.96
*SsDof20-1*	*SsDof20-3*	Segmental duplication	0.0025	0.0111	0.2234	0.91
*SsDof20-1*	*SsDof20-4*	Segmental duplication	0.0132	0.0326	0.4038	2.67
*SsDof20-2*	*SsDof20-3*	Tandem duplication	0.0127	0.019	0.6674	1.56
*SsDof20-2*	*SsDof20-4*	Segmental duplication	0.0216	0.0363	0.5954	2.97
*SsDof20-3*	*SsDof20-4*	Segmental duplication	0.0138	0.0225	0.6159	1.84
*SsDof22-3*	*SsDof22-1*	Segmental duplication	0.0013	0.0074	0.1737	0.61
*SsDof22-3*	*SsDof22-4*	Segmental duplication	0.0052	0.0037	1.3941	0.3
*SsDof23-1*	*SsDof23-2*	Segmental duplication	0.0083	0.018	0.4626	1.48
*SsDof27-2*	*SsDof27-3*	Segmental duplication	0.013	0.0232	0.5592	1.9
*SsDof27-2*	*SsDof27-4*	Segmental duplication	0.0189	0.0275	0.6851	2.26
*SsDof27-3*	*SsDof27-4*	Segmental duplication	0.0097	0.0435	0.2236	3.56
*SsDof28-1*	*SsDof28-2*	Segmental duplication	0.0697	0.1019	0.6837	8.35
*SsDof28-1*	*SsDof28-4*	Segmental duplication	0.009	0.0187	0.4786	1.53
*SsDof28-4*	*SsDof28-2*	Segmental duplication	0.1237	0.1903	0.6502	15.6

### *Cis*-elements analysis of *SsDof* genes in sugarcane

**Fig 5:**
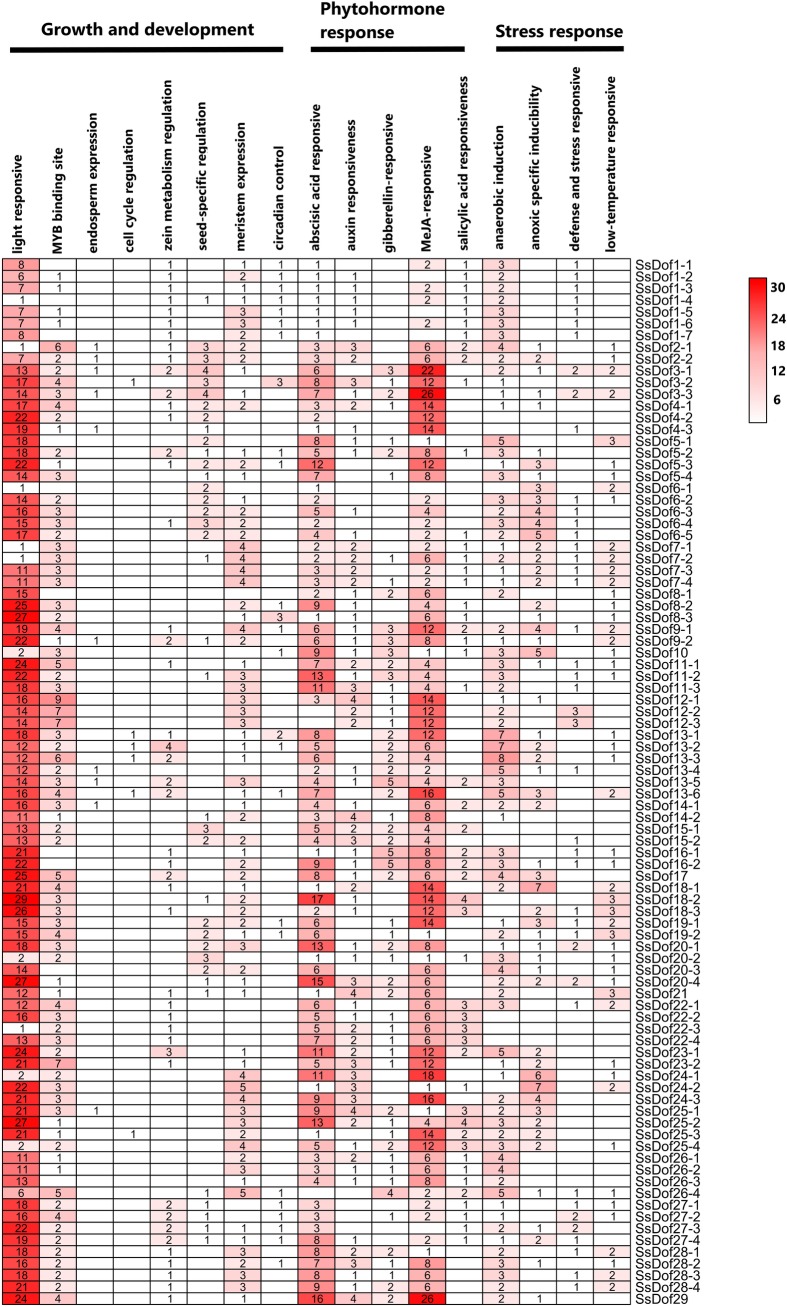
*Cis*-elements distribution in promoters of *SsDof* genes. The numbers of *cis*-elements of *SsDof* genes for growth and development, phytohormone response, and stress response are indicated with different color boxes.

We checked the *cis*-elements of *SsDof* genes and collected the *cis*-elements for growth and development, plant hormones and abiotic stresses responses in plants (Fig 5). For plant growth and development, the most frequent *cis*-elements identified were G-box and Sp1elements, which are related to light responses. The ABRE elements and TGACG motifs and CGTCA motifs were the most frequent elements for plant hormones-related *cis*-elements. For abiotic stress responses, ARE element included the most numbers of elements (S4 Table). Additionally, the promoter of *SsDof13* contained most *cis*-elements of MYB binding site motifs. The promoter of *SsDof20* contained most ABA responsive *cis*-elements, whereas *SsDof3* contained most MeJA responsive elements and *SsDof13* contained most anaerobic induction elements (Fig 5).

The *cis*-elements of *SsDofs’* alleles distributed differently (Fig 5). The number of *cis*-elements for plant hormone responses distributed differently in *SsDofs’* alleles. For example, the numbers of MeJA-responsive elements were different between alleles of *SsDof3*, *SsDof4*, *SsDof5*, *SsDof6*, *SsDof9*, *SsDof13* and *SsDof25*. The numbers of gibberellin response elements were detected differently in alleles of *SsDof3*, *SsDof4*, *SsDof5* and *SsDof26*. And the numbers of auxin responsive elements were also detected differently between the alleles of *SsDof2*, *SsDof3*, *SsDof15* and *SsDof28*. In addition, the number of *cis*-elements for plant growth and development distributed differently in some *SsDofs’* alleles, such as the numbers of light responses elements between alleles of *SsDof1*, *SsDof3*, *SsDof5*, *SsDof13*, *SsDof18*, *SsDof20* and *SsDof27*, the numbers of the MYB binding site elements between alleles of *SsDof3*, *SsDof4*, *SsDof5*, *SsDof13* and *SsDof22*.

### Expression profiling of *SsDof* genes

**Fig 6:**
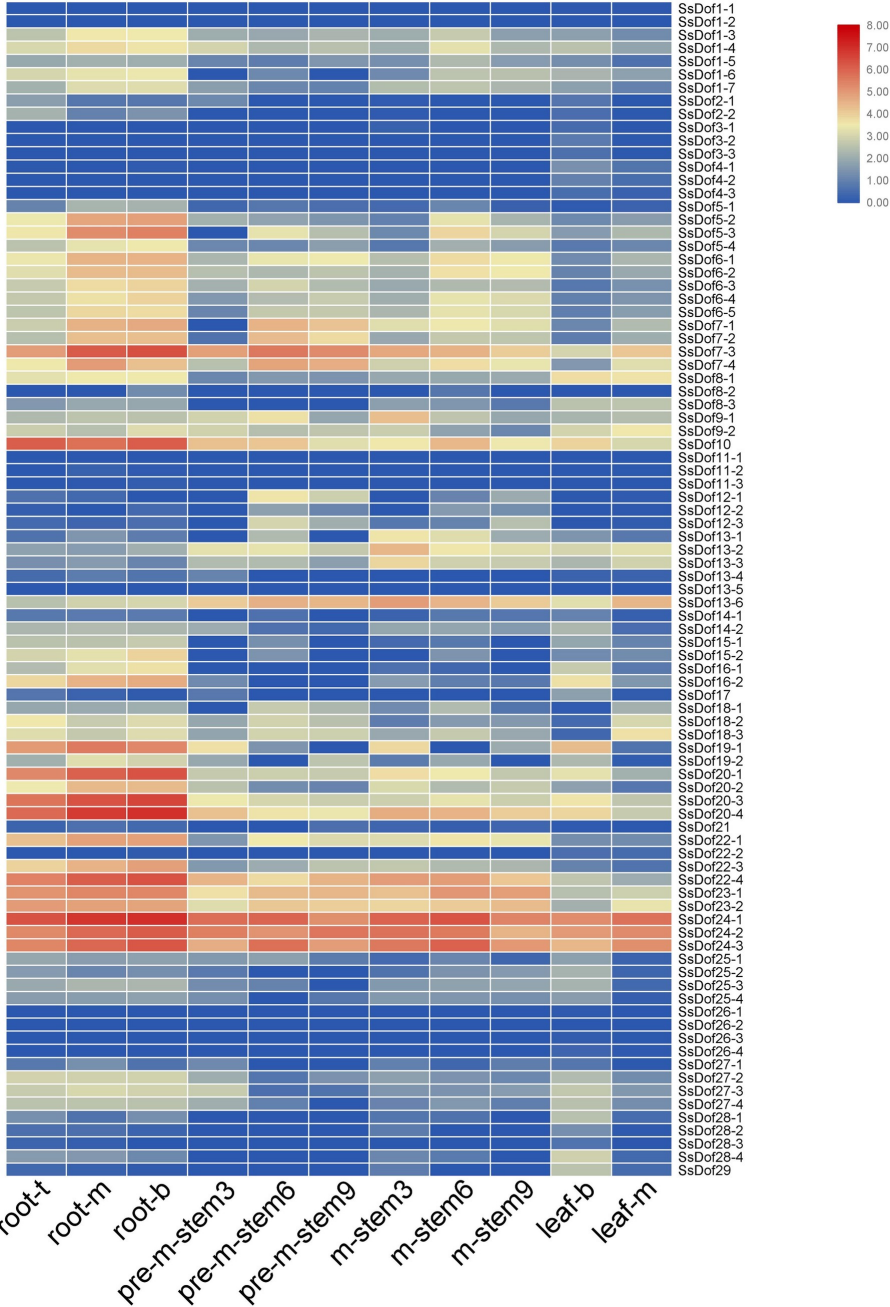
Expression profiling of *SsDof* genes in sugarcane. The root samples were obtained from root in seedling stage (45 days old), including the top of root (below the root hair, root-t), the middle of root (root-m) and the base of the root (root-b). The stem and leaf samples were from 9 months old premature internode (pre-m-stem3, pre-m-stem6 and pre-m-stem9), 12 months old mature internode (m-stem3, m-stem6 and m-stem9) and leaf (leaf-b, leaf-m and leaf-u).

To investigate the expression profiling of *SsDofs*, we examined the transcription levels of *SsDofs* in different tissues and stages, including root in seedling stage, stem in premature and mature stage, and leaf in mature stage (Fig 6). Among these *SsDofs*, *SsDof1-2*, *SsDof26-2* and *SsDof13-5* was not expressed in all samples, which may have special temporal expression patterns not examined in our libraries. And forty *SsDofs* (44.9%) were expressed in all samples. *SsDof7*, *SsDof23* and *SsDof24* had a high expression in all detected tissues. The expression of *SsDof1-1*, *SsDof3-2*, *SsDof3-3*, *SsDof4-2*, *SsDof4-3*, *SsDof11-1*, *SsDof26-1*, *SsDof26-3* and *SsDof26-4* were only detected in leaves, indicating that they may be involved in leaf development. Additionally, *SsDof4-1*, *SsDof11-2*, *SsDof11-3*, *SsDof22-2* and *SsDof28-3* only expressed in roots and leaves, indicating that these *SsDofs* may be associated with leaf and root development.

Expression profiling of *SsDofs’* alleles displayed differently. Some alleles of *SsDof* genes displayed similar expression profiling, such as the alleles of *SsDof3*, *SsDof6*, *SsDof17*, *SsDof24* and *SsDof26*. However, the expression patterns were different for many *SsDofs’* alleles. For example, *SsDof1-3*, *SsDof1-4*, *SsDof1-6* and *SsDof1-7* showed comparatively higher levels of expression in root, while *SsDof1-1* and *SsDof1-2* showed low expressions. *SsDof7-3* had a high expression in all detected samples, while *SsDof7-1*, *SsDof7-2* and *SsDof7-4* had comparatively lower levels of expression in all detected tissues (Fig 6).

**Fig 7:**
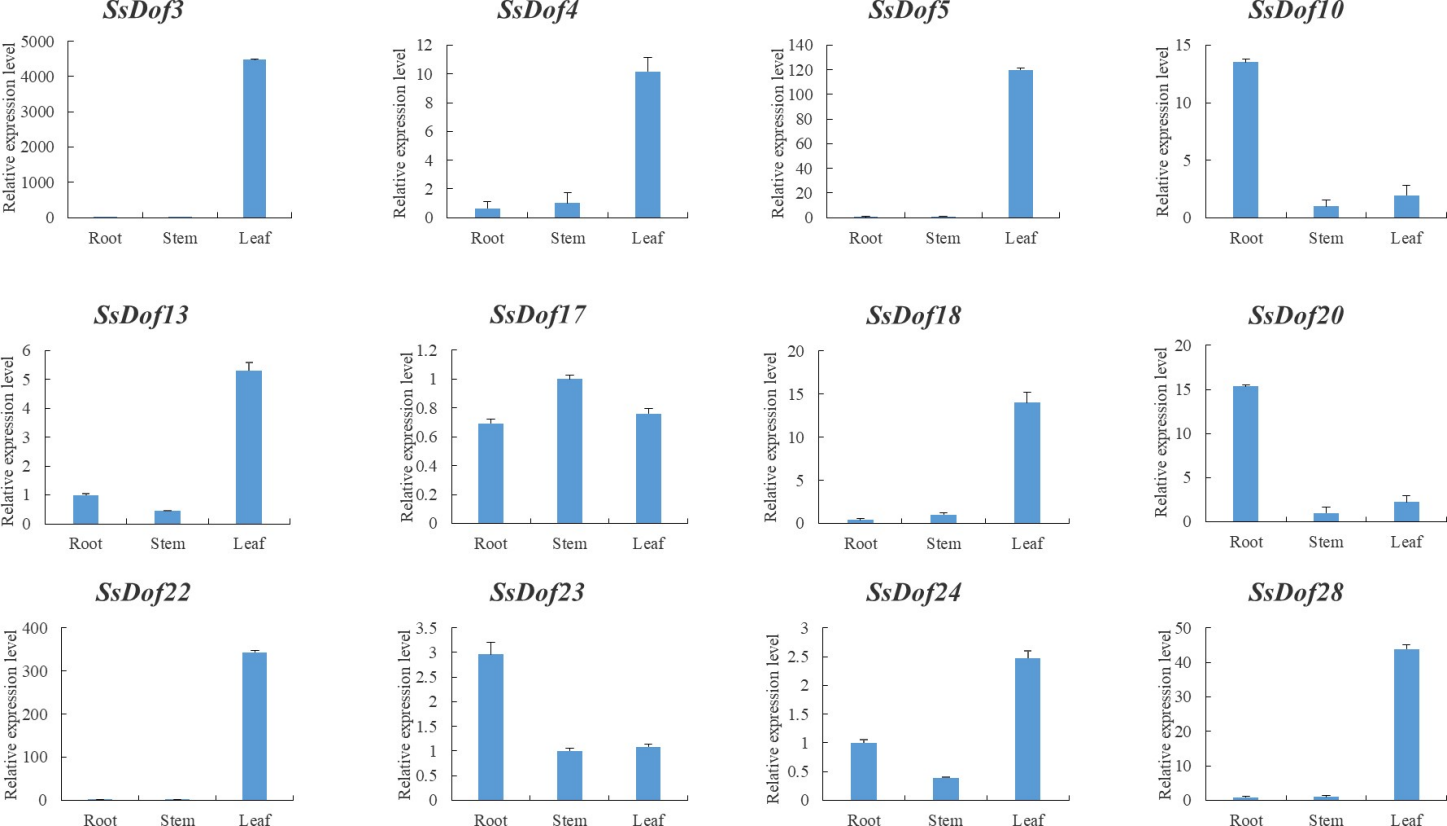
Expression levels of *SsDof* genes at the seedling stage by qRT-PCR. The tissue samples were obtained from root, stem and leaf in seedling stage (45 days old). The standard deviation was indicated with the vertical bars.

In order to verify the transcriptome data, we carried out the quantitative real-time PCR experiments. *SsDof10*, *SsDof20* and *SsDof23* showed comparatively higher levels of expression in root, while *SsDof3*, *SsDof4*, *SsDof5*, *SsDof13*, *SsDof18*, *SsDof22*, *SsDof24* and *SsDof28* showed relatively higher levels of expression in leaf (Fig 7). All the 12 *SsDofs* showed a very low level of expression in stem except *SsDof17* and *SsDof23*. Our results were identical to the expression profiling of *SsDof* genes detected by RNA-Seq.

### Expression profiling of *SsDof* genes responding to plant hormones

**Fig 8:**
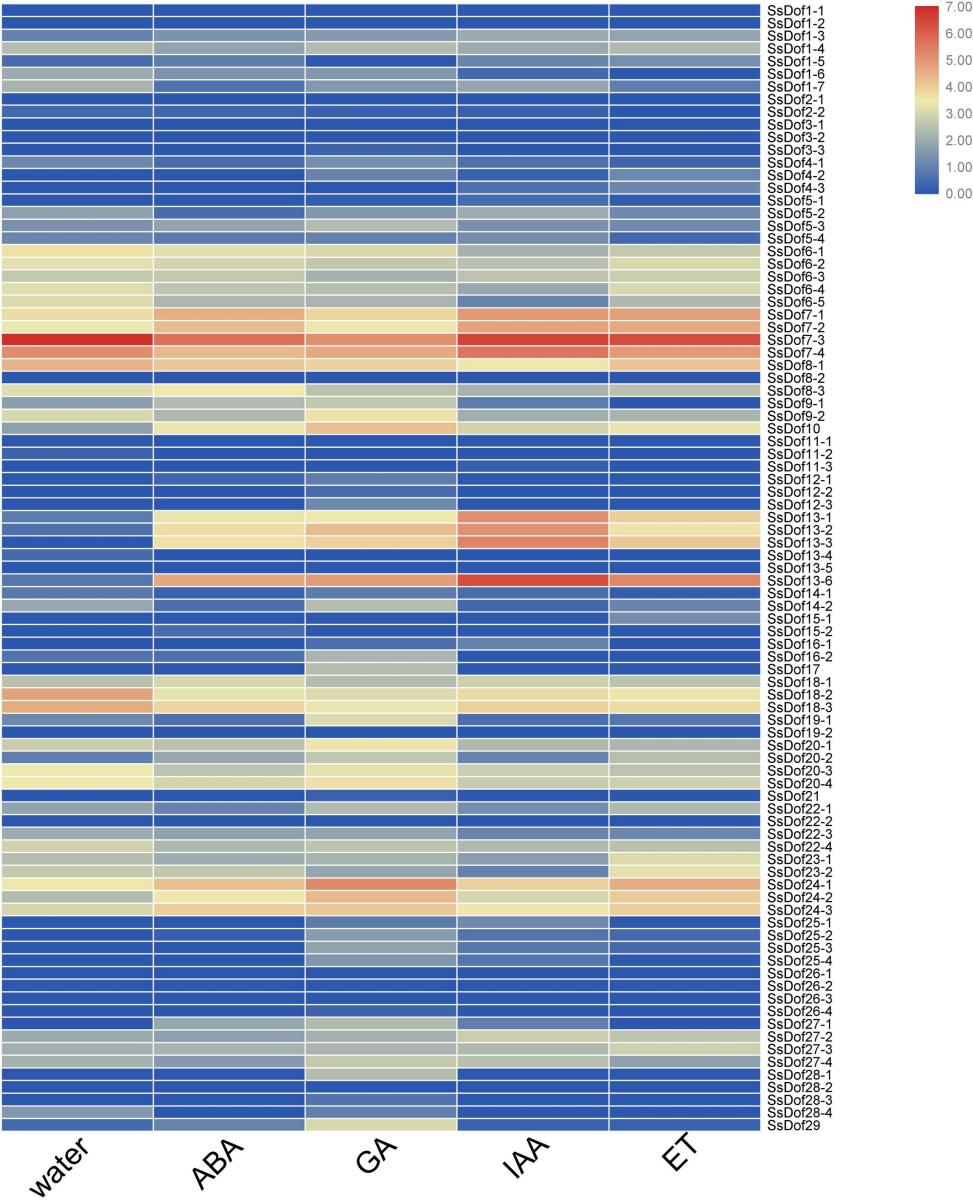
Expression profiling of *SsDof* genes responding to plant hormones based on RNA-Seq. The whole sugarcane seedlings (45 days old) were subjected to four plant hormones (ABA, GA, Auxin and Ethylene) and the leaves were collected at 24 h after treatments. The heatmap was generated by TBtools with the FPKM values of each tissue sample.

To investigate the expression profiling of *SsDof* genes responding to plant hormones, we examined their transcription levels under four plant hormones treatments (ABA, GA, Auxin and Ethylene). As shown in Fig 8, *SsDof7* and *SsDof10* were up-regulated under ABA treatment, but *SsDof18* were down-regulated. After GA treatment, *SsDof13* and *SsDof24* were up-regulated, but *SsDof18* were down-regulated. The expression of *SsDof10*, *SsDof13* and *SsDof24* increased after IAA treatment, but the transcription levels of *SsDof8* and *SsDof18* reduced. Under ET treatment, *SsDof7* were up-regulated whereas *SsDof9* and *SsDof18* were down-regulated. Interestingly, after four plant hormones treatment, *SsDof10* and *SsDof13* were up-regulated whereas *SsDof18* were down-regulated.

In addition, some alleles of *SsDof* genes displayed similar expression profiling, such as *SsDof2-1*, *SsDof2-2*, *SsDof3-1*, *SsDof3-2*, *SsDof3-3*, *SsDof11-1*, *SsDof11-2* and *SsDof11-3*. However, some of *SsDofs’* alleles showed opposite expression pattern. For example, *SsDof7-1* and *SsDof7-2* were up-regulated after ABA treatment, while *SsDof7-3* and *SsDof7-4* were down-regulated.

### Expression profiling of *SsDof* genes responding to abiotic stresses

**Fig 9:**
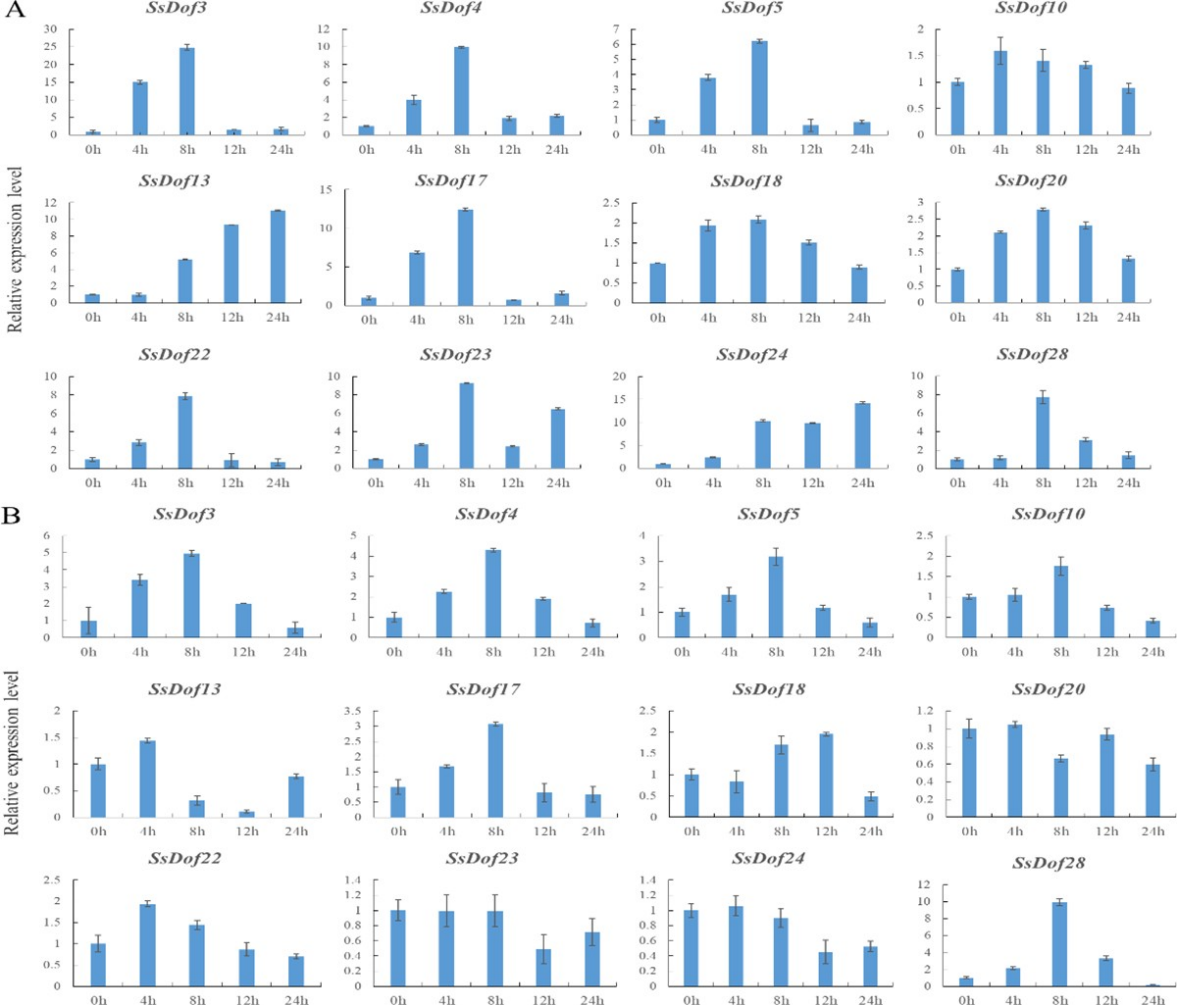
Expression levels of *SsDof* genes under cold and heat treatments by qRT-PCR. The tissue samples were obtained from leave in seeding stage (45 days old). The standard deviation was indicated with the vertical bars. (A). The sugarcane seedlings were performed with 4°C for 4, 8, 12 and 24 h respectively. (B). The sugarcane seedlings were performed with 38°C for 4, 8, 12 and 24 h respectively.

**Fig 10:**
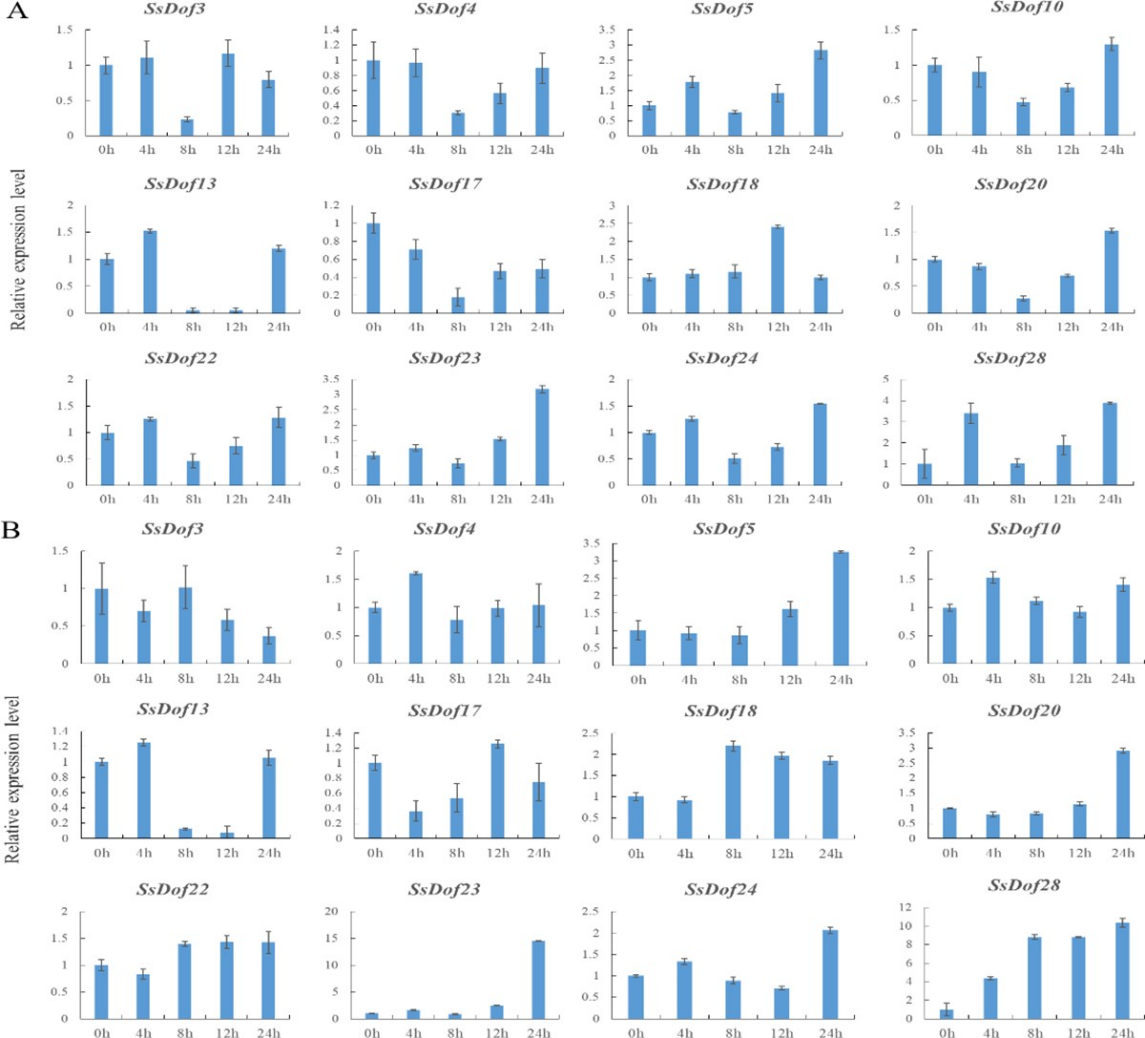
Expression levels of *SsDof* genes under salt and drought treatments by qRT-PCR. The tissue samples were obtained from leaf in seeding stage (45 days old). The standard deviation was indicated with the vertical bars. (A). The sugarcane seedlings were performed with 100 mM NaCl for 4, 8, 12 and 24 h respectively. (B). The sugarcane seedlings were performed with 15% PEG6000 for 4, 8, 12 and 24 h respectively.

12 *SsDof* members were selected from 29 sugarcane *SsDof* genes to investigate the expression profiling under various abiotic stresses. Then we conducted qRT-PCR experiments to observe their expression patterns after four treatments (4°C, 38°C, NaCl, PEG). As shown in Figs 9 and 10, *SsDof5* and *SsDof28* were obviously responding to all four treatments. The expression of *SsDof5*, *SsDof10*, *SsDof18* and *SsDof28* increased after these four treatments. All 12 *SsDof* genes were induced after cold treatment whereas *SsDof3*, *SsDof4*, *SsDof5*, *SsDof17* and *SsDof28* were induced after heat treatment. The transcription levels of *SsDof4* and *SsDof17* decreased after salt treatment. After different treatments, some *SsDof* genes showed opposite expression patterns. For example, *SsDof17* was obviously up-regulated after cold and heat treatment whereas was down-regulated by salt treatment.

## Discussion

Gene expression profiles provide valuable clues for gene function. In Arabidopsis, *AtDof5*.*8* was involved in processes of vascular development [40,41]. *SsDof23* is orthologous to *AtDof5*.*8* and had a high expression in roots and stem, indicating that *SsDof23* may contain some similar functions in the development of sugarcane vascular tissues. Moreover, *SsDof1*, *SsDof8*, *SsDof25* and *SsDof27* are orthologous to *AtDof5*.*7*, which had been confirmed to control the differentiation of guard cells by controlling the transcription levels of genes [42]. Interestingly, the expression profiling of most *SsDofs*’ alleles displayed differently, such as the alleles of *SsDof1*, *SsDof1-4* had a high expression in roots, whereas *SsDof1-1* and *SsDof1-5* had a low expression in roots. And the promoter regions of *SsDof1-4*, *SsDof1-1* and *SsDof1-5* contained different numbers of *cis*-elements for plant growth and development. These results indicated that allele specific expression of *SsDof* genes may be associated with *cis*-elements for plant growth and development.

There are previous studies about *Dof* genes in response to plant hormones. In potato, *StDof* genes showed either ABA-independent or ABA-dependent expression profiling [43]. In tobacco, *NtBBF1*was reported to facilitate the auxin-inducible gene expression [44]. In our study, *SsDof10*, *SsDof13-1*, *SsDof13-2*, *SsDof13-3*, *SsDof13-6* and *SsDof24-1* were up-regulated under four plant hormone treatments, whereas *SsDof18-2* and *SsDof18-3* were down-regulated, indicating these *SsDofs* may play important roles in response to phytohormones. Interestingly, *SsDof7-1* and *SsDof7-2* were up-regulated after ABA treatment, while *SsDof7-3* and *SsDof7-4* were repressed. Meanwhile, *SsDof7-3* and *SsDof7-4* had more abscisic acid responsive elements than *SsDof7-1* or *SsDof7-2* in their promoter regions. Our results suggested that allele specific expression of *SsDof* genes responding to hormones may be associated with *cis*-elements for plant hormones.

*Cis*-elements play critical roles in regulating phytohormones and abiotic stresses responses in plants [45,46]. The most *cis*-elements we have identified are those associated with light responsive, indicating light signals may play critical roles in transcriptional regulation of *SsDofs* in *S*. *spontaneum* AP85-441. Moreover, we also identified numbers of *cis*-elements about plant hormones and abiotic stresses in promoter regions of *SsDofs*. Meanwhile, most *SsDofs* were responsive to phytohormones and abiotic stresses detected by our data. These results suggested that *SsDof* genes may be involved in responding to phytohormones and abiotic stresses.

*Dof* genes have been reported to be associate with abiotic stresses responses. In Arabidopsis, the transcription levels of *AtDof1*.*1* was up-regulated for three times under MeJA treatment, damaging the plant tissues [47]. In Chinese cabbage, many *BraDof* genes were induced obviously after cold, heat, salt and drought stresses. In tomato, *SlCDF1-5* was obviously up-regulated after osmotic, cold, heat and salt treatments. Similar to previous researches, many *SsDof* genes were induced or repressed under cold, heat, salt and drought stresses, indicating that *SsDof* genes may be involved in responding to abiotic stresses. Interestingly, those *SsDof* genes induced were always detected about 4 hours after abiotic stresses treatments, indicating *SsDofs’* expression increased immediately under cold and heat stresses. Under diverse treatments, some *SsDof* genes presented reverse expression patterns. For instance, *SsDof17* was significantly induced by cold and heat treatment, whereas was repressed by salt treatment. Our study demonstrated that *SsDof* genes may play important roles in responding to various abiotic stresses in sugarcane.

Gene, genome, and segmental duplications are reported to be associate with genetic novelty [48–50]. The sugarcane genome was identified to undergo two WGD events after divergence from its closest relative and detailed analysis of the genome showed duplications in other gene families [51–56]. The duplications of *SsDofs* in sugarcane originated from approximately 0.21 Mya to 15.60 Mya, which indicated the duplications of *SsDofs* in sugarcane took place prior and after the divergence of sugarcane and sorghum. Moreover, we identified forty-nine pairs of duplicated *SsDof* gene pairs, including forty-six pairs of segmentally duplicated genes and three pairs of tandemly duplicated genes. This result suggested that segmental duplications are predominant in the evolution of *SsDof* in sugarcane.

## Conclusions

We performed a comprehensive and systematic analysis to investigate the *Dof* genes in sugarcane genome and 29 *SsDof* genes were identified. Those *SsDof* genes were divided into five groups, with similar gene structures and motif patterns in the same group. Forty-nine pairs of duplicated *SsDof* genes were identified in sugarcane chromosomes. The duplications of *SsDof* genes originated from approximately 0.21 Mya to 15.60 Mya. *Cis*-element analysis suggested that the functions of *SsDofs* were involved in growth and development, hormone and abiotic stress responses in sugarcane. Expression patterns indicated that *SsDof* genes are crucial in sugarcane growth and development. The transcription levels of *SsDofs* under plant hormone treatments indicated that different alleles may play different roles in response to plant hormones. *SsDofs’* expression profiling under four abiotic stresses indicated that they are involved in abiotic stress responses in sugarcane. This work provides a foundation for further functional analysis of *SsDof* genes in sugarcane.

## Supporting information

S1 Table List of *SsDof* genes identified in this study. (XLSX)

S2 Table The full length Dof protein sequences in *Arabidopsis thaliana* (*AtDof1*.*1–5*.*8*) and *Sorghum bicolor* (*SbDof1-28*) used in phylogenetic tree construction. (DOCX)

S3 Table Duplicated *Dof* genes in *Saccharum spontaneum*. (XLSX)

S4 Table Total *cis*-elements of *SsDof* genes. (DOCX)

S5 Table RNA-seq data of *SsDof* genes. (XLSX)

S6 Table The primers of *SsDof* genes in this study. (DOCX)
