# Supplementary material for: Allele specific expression of Dof genes responding to hormones and abiotic stresses in sugarcane
Source: PLoS One. 2020 Jan 16;15(1):e0227716. doi: 10.1371/journal.pone.0227716 (PMC6964845; doi:10.1371/journal.pone.0227716)
Supplement: S2 Table — (DOCX) [file pone.0227716.s002.docx]

**The full length Dof protein sequences in *Arabidopsis thaliana* (*AtDof1.1-5.8*) and *Sorghum bicolor* (*SbDof1-28*) used in phylogenetic tree construction.**

>AtDof1.1

MGGSMAERARQANIPPLAGPLKCPRCDSSNTKFCYYNNYNLTQPRHFCKGCRRYWTQGGALRNVPVGGGCRRNNKKGKNGNLKSSSSSSKQSSSVNAQSPSSGQLRTNHQFPFSPTLYNLTQLGGIGLNLAATNGNNQAHQIGSSLMMSDLGFLHGRNTSTPMTGNIHENNNNNNNENNLMASVGSLSPFALFDPTTGLYAFQNDGNIGNNVGISGSSTSMVDSRVYQTPPVKMEEQPNLANLSRPVSGLTSPGNQTNQYFWPGSDFSGPSNDLL

>AtDof1.2

MLPYIGHNSYQQHQFPLPEMEIPEKWKLSYEQEAITAPACPRCASSNTKFCYYNNYSLSQPRYFCKGCRRYWTKGGSLRNIPVGGGCRKRSRSRQNSHKRFGRNENRPDGLINQDDGFQSSPPGSDIDLAAVFAQYVTDRSPSSTDNTTGSDQDSPITTTTHALESLSWDICQETDVDLGFYGEFNNLTQKTKEDQEVFGQFLQEDREEIFEFQGLLDDKEIQEILECSFSEEPDQLVSQGSFMINGDNWSSTDLTRFGI

>AtDof1.3

MWLSHLFMSLSKLTCNFSIFSVFMACGSIGMSQVRDTPVKLFGWTITPVSHDPYSSSSHVLPDSSSSSSSSSLSLRPHMMNNQSVTDNTSLKLSSNLNNESKETSENSDDQHSEITTITSEEEKTTELKKPDKILPCPRCNSADTKFCYYNNYNVNQPRHFCRKCQRYWTAGGSMRIVPVGSGRRKNKGWVSSDQYLHITSEDTDNYNSSSTKILSFESSDSLVTERPKHQSNEVKINAEPVSQEPNNFQGLLPPQASPVSPPWPYQYPPNPSFYHMPVYWGCAIPVWSTLDTSTCLGKRTRDETSHETVKESKNAFERTSLLLESQSIKNETSMATNNHVWYPVPMTREKTQEFSFFSNGAETKSSNNRFVPETYLNLQANPAAMARSMNFRESI

>AtDof1.4

MPIISSPNTNPLASMQSKNMIVASSHQQQQQQQPQQPQPQLKCPRCDSSNTKFCYYNNYSLSQPRHFCKACKRYWTRGGTLRNVPVGGSYRKNKRVKRPSTATTTTASTVSTTNSSSPNNPHQISHFSSMNHHPLFYGLSDHMSSCNNNLPMIPSRFSDSSKTCSSSGLESEFLSSGFSSLSALGLGLPHQMSHDHTINGSFINNSTTNKPFLLSGLFGSSMSSSSTLLQHPHKPMNNGGDMLGQSHLQTLASLQDLHVGGNNEDMKYKEGKLDQISGNINGFMSSSSSLDPSNYNNMWNNASVVNGAWLDPTNNNVGSSLTSLI

>AtDof1.5

MATQDSQGIKLFGKTITFNANITQTIKKEEQQQQQQPELQATTAVRSPSSDLTAEKRPDKIIPCPRCKSMETKFCYFNNYNVNQPRHFCKGCQRYWTAGGALRNVPVGAGRRKSKPPGRVGGFAELLGAATGAVDQVELDALLVEEWRAATASHGGFRHDFPVKRLRCYTDGQSC

>AtDof1.6

MPSEPNQTRPTRVQPSTAAYPPPNLAEPLPCPRCNSTTTKFCYYNNYNLAQPRYYCKSCRRYWTQGGTLRDVPVGGGTRRSSSKRHRSFSTTATSSSSSSSVITTTTQEPATTEASQTKVTNLISGHGSFASLLGLGSGNGGLDYGFGYGYGLEEMSIGYLGDSSVGEIPVVDGCGGDTWQIGEIEGKSGGDSLIWPGLEISMQTNDVK

>AtDof1.7

MQDLTSAAAYYHQSMMMTTAKQNQPELPEQEQLKCPRCDSPNTKFCYYNNYNLSQPRHFCKNCRRYWTKGGALRNIPVGGGTRKSNKRSGSSPSSNLKNQTVAEKPDHHGSGSEEKEERVSGQEMNPTRMLYGLPVGDPNGASFSSLLASNMQMGGLVYESGSRWLPGMDLGLGSVRRSDDTWTDLAMNRMEKN

>AtDof1.8

MDTAKWPQEFVVKPMNEIVTNTCLKQQSNPPSPATPVERKARPEKDQALNCPRCNSLNTKFCYYNNYSLTQPRYFCKDCRRYWTAGGSLRNIPVGGGVRKNKRSSSNSSSSSPSSSSSSKKPLFANNNTPTPPLPHLNPKIGEAAATKVQDLTFSQGFGNAHEVKDLNLAFSQGFGIGHNHHSSIPEFLQVVPSSSMKNNPLVSTSSSLELLGISSSSASSNSRPAFMSYPNVHDSSVYTASGFGLSYPQFQEFMRPALGFSLDGGDPLRQEEGSSGTNNGRPLLPFESLLKLPVSSSSTNSGGNGNLKENNDEHSDHEHEKEEGEADQSVGFWSGMLSAGASAAASGGSWQ

>AtDof1.10

MSKSRDTEIKLFGRTITSLLDVNCYDPSSLSPVHDVSSDPSKEDSSSSSSSCSPTIGPIRVPVKKSEQESNKFKDPYILSDLNEPPKAVSEISSPRSSKNNCDQQSEITTTTTTSTTSGEKSTALKKPDKLIPCPRCESANTKFCYYNNYNVNQPRYFCRNCQRYWTAGGSMRNVPVGSGRRKNKGWPSSNHYLQVTSEDCDNNNSGTILSFGSSESSVTETGKHQSGDTAKISADSVSQENKSYQGFLPPQVMLPNNSSPWPYQWSPTGPNASFYPVPFYWGCTVPIYPTSETSSCLGKRSRDQTEGRINDTNTTITTTRARLVSESLRMNIEASKSAVWSKLPTKPEKKTQGFSLFNGFDTKGNSNRSSLVSETSHSLQANPAAMSRAMNFRESMQQ

>AtDof2.1

MDPEQEISNETLETILVSSTKGSNNNNKKMEEEMKKKVSRGELGGEAQNCPRCESPNTKFCYYNNYSLSQPRYFCKSCRRYWTKGGTLRNVPVGGGCRRNKRSSSSAFSKNNNNKSINFHTDPLQNPLITGMPPSSFGYDHSIDLNLAFATLQKHHLSSQATTPSFGFGGDLSIYGNSTNDVGIFGGQNGTYNNSLCYGFMSGNGNNNQNEIKMASTLGMSLEGNERKQENVNNNNNNSENPSKVFWGFPWQMTGDSAGVVPEIDPGRESWNGMVSSWNNGLLNTPLV

>AtDof2.2

MVFSSVSSFLDPPINWPQSANPNNHPHHHQLQENGSLVSGHHQVLSHHFPQNPNPNHHHVETAAATTVDPSSLNGQAAERARLAKNSQPPEGALKCPRCDSANTKFCYFNNYNLTQPRHFCKACRRYWTRGGALRNVPVGGGCRRNKKGKSGNSKSSSSSQNKQSTSMVNATSPTNTSNVQLQTNSQFPFLPTLQNLTQLGGIGLNLAAINGNNGGNGNTSSSFLNDLGFFHGGNTSGPVMGNNNENNLMTSLGSSSHFALFDRTMGLYNFPNEVNMGLSSIGATRVSQTAQVKMEDNHLGNISRPVSGLTSPGNQSNQYWTGQGLPGSSSNDHHHQHLM

>AtDof2.3

MATQDSQGIKLFGKTIAFNTRTIKNEEETHPPEQEATIAVRSSSSSDLTAEKRPDKIIACPRCKSMETKFCYFNNYNVNQPRHFCKGCHRYWTAGGALRNVPVGAGRRKSKPPGRVVVGMLGDGNGVRQVELINGLLVEEWQHAAAAAHGSFRHDFPMKRLRCYSDGQSC

>AtDof2.4

MVFSSIQAYLDSSNWQQAPPSNYNHDGTGASANGGHVLRPQLQPQQQPQQQPHPNGSGGGGGGGGGSIRAGSMVDRARQANVALPEAALKCPRCESTNTKFCYFNNYSLTQPRHFCKTCRRYWTRGGALRNVPVGGGCRRNRRTKSNSNNNNNSTATSNNTSFSSGNASTISTILSSHYGGNQESILSQILSPARLMNPTYNHLGDLTSNTKTDNNMSLLNYGGLSQDLRSIHMGASGGSLMSCVDEWRSASYHQQSSMGGGNLEDSSNPNPSANGFYSFESPRITSASISSALASQFSSVKVEDNPYKWVNVNGNCSSWNDLSAFGSSR

>AtDof2.5

MDATKWTQGFQEMMNVKPMEQIMIPNNNTHQPNTTSNARPNTILTSNGVSTAGATVSGVSNNNNNTAVVAERKARPQEKLNCPRCNSTNTKFCYYNNYSLTQPRYFCKGCRRYWTEGGSLRNVPVGGSSRKNKRSSSSSSSNILQTIPSSLPDLNPPILFSNQIHNKSKGSSQDLNLLSFPVMQDQHHHHVHMSQFLQMPKMEGNGNITHQQQPSSSSSVYGSSSSPVSALELLRTGVNVSSRSGINSSFMPSGSMMDSNTVLYTSSGFPTMVDYKPSNLSFSTDHQGLGHNSNNRSEALHSDHHQQGRVLFPFGDQMKELSSSITQEVDHDDNQQQKSHGNNNNNNNSSPNNGYWSGMFSTTGGGSSW

>AtDof3.1

MQDPAAYYQTMMAKQQQQQQPQFAEQEQLKCPRCDSPNTKFCYYNNYNLSQPRHFCKSCRRYWTKGGALRNVPVGGGSRKNATKRSTSSSSSASSPSNSSQNKKTKNPDPDPDPRNSQKPDLDPTRMLYGFPIGDQDVKGMEIGGSFSSLLANNMQLGLGGGGIMLDGSGWDHPGMGLGLRRTEPGNNNNNPWTDLAMNRAEKN

>AtDof3.2

MDYSSMHQNVMGVSSCSTQDYQNQKKPLSATRPAPPEQSLRCPRCDSTNTKFCYYNNYSLSQPRYFCKSCRRYWTKGGILRNIPIGGAYRKHKRSSSATKSLRTTPEPTMTHDGKSFPTASFGYNNNNISNEQMELGLAYALLNKQPLGVSSHLGFGSSQSPMAMDGVYGTTSHQMENTGYAFGNGGGGMEQMATSDPNRVLWGFPWQMNMGGGSGHGHGHVDQIDSGREIWSSTVNYINTGALL

>AtDof3.3

MMMETRDPAIKLFGMKIPFPSVFESAVTVEDDEEDDWSGGDDKSPEKVTPELSDKNNNNCNDNSFNNSKPETLDKEEATSTDQIESSDTPEDNQQTTPDGKTLKKPTKILPCPRCKSMETKFCYYNNYNINQPRHFCKACQRYWTAGGTMRNVPVGAGRRKNKSSSSHYRHITISEALEAARLDPGLQANTRVLSFGLEAQQQHVAAPMTPVMKLQEDQKVSNGARNRFHGLADQRLVARVENGDDCSSGSSVTTSNNHSVDESRAQSGSVVEAQMNNNNNNNMNGYACIPGVPWPYTWNPAMPPPGFYPPPGYPMPFYPYWTIPMLPPHQSSSPISQKCSNTNSPTLGKHPRDEGSSKKDNETERKQKAGCVLVPKTLRIDDPNEAAKSSIWTTLGIKNEAMCKAGGMFKGFDHKTKMYNNDKAENSPVLSANPAALSRSHNFHEQI

>AtDof3.4

MPTSDSGEPRRIAMKPNGVTVPISDQQEQLPCPRCDSSNTKFCYYNNYNFSQPRHFCKACRRYWTHGGTLRDVPVGGGTRKSAKRSRTCSNSSSSSVSGVVSNSNGVPLQTTPVLFPQSSISNGVTHTVTESDGKGSALSLCGSFTSTLLNHNAAATATHGSGSVIGIGGFGIGLGSGFDDVSFGLGRAMWPFSTVGTATTTNVGSNGGHHAVPMPATWQFEGLESNAGGGFVSGEYFAWPDLSITTPGNSLK

>AtDof3.5

MERAEALTSSFIWRPNANANAEITPSCPRCGSSNTKFCYYNNYSLTQPRYFCKGCRRYWTKGGSLRNVPVGGGCRKSRRPKSSSGNNTKTSLTANSGNPGGGSPSIDLALVYANFLNPKPDESILQENCDLATTDFLVDNPTGTSMDPSWSMDINDGHHDHYINPVEHIVEECGYNGLPPFPGEELLSLDTNGVWSDALLIGHNHVDVGVTPVQAVHEPVVHFADESNDSTNLLFGSWSPFDFTADG

>AtDof3.6

MVFSSLPVNQFDSQNWQQMISILVFFSTSRLFKKLFLVDKNLFSCLLQGLMYNVFLTGLIFSLQGNQHQLECVTTDQNPNNYLRQLSSPPTSQVAGSSQARVNSMVERARIAKVPLPEAALNCPRCDSTNTKFCYFNNYSLTQPRHFCKTCRRYWTRGGSLRNVPVGGGFRRNKRSKSRSKSTVVVSTDNTTSTSSLTSRPSYSNPSKFHSYGQIPEFNSNLPILPPLQSLGDYNSSNTGLDFGGTQISNMISGMSSSGGILDAWRIPPSQQAQQFPFLINTTGLVQSSNALYPLLEGGVSATQTRNVKAEENDQDRGRDGDGVNNLSRNFLGNININSGRNEEYTSWGGNSSWTGFTSNNSTGHLSF

>AtDof3.7

MDATKWTQGFQEMINVKPMEQMISSTNNNTPQQQPTFIATNTRPNATASNGGSGGNTNNTATMETRKARPQEKVNCPRCNSTNTKFCYYNNYSLTQPRYFCKGCRRYWTEGGSLRNVPVGGSSRKNKRSSTPLASPSNPKLPDLNPPILFSSQIPNKSNKDLNLLSFPVMQDHHHHGMSHFFHMPKIENNNTSSSIYASSSPVSALELLRSNGVSSRGMNTFLPGQMMDSNSVLYSSLGFPTMPDYKQSNNNLSFSIDHHQGIGHNTINSNQRAQDNNDDMNGASRVLFPFSDMKELSSTTQEKSHGNNTYWNGMFSNTGGSSW

>AtDof4.1

MDHHQYHHHDQYQHQMMTSTNNNSYNTIVTTQPPPTTTTMDSTTATTMIMDDEKKLMTTMSTRPQEPRNCPRCNSSNTKFCYYNNYSLAQPRYLCKSCRRYWTEGGSLRNVPVGGGSRKNKKLPFPNSSTSSSTKNLPDLNPPFVFTSSASSSNPSKTHQNNNDLSLSFSSPMQDKRAQGHYGHFSEQVVTGGQNCLFQAPMGMIQFRQEYDHEHPKKNLGFSLDRNEEEIGNHDNFVVNEEGSKMMYPYGDHEDRQQHHHVRHDDGNKKREGGSSNELWSGIILGGDSGGPTW

>AtDof4.2

MNNLNVFTNEDNEMNVMPPPRVCPRCYSDQTRFSYFNNNKKSQPRYKCKNCCRCWTHGGVLRNIPVTGICDKSNLPKIDQSSVSQMILAEIQQGNHQPFKKFQENISVSVSSSSDVSIVGNHFDDLSELHGITNSTPIRSFTMDRLDFGEESFQQDLYDVGSNDLIGNPLINQSIGGYVDNHKDEHKLQFEYES

>AtDof4.3

MDNFNVVANEDNQVNDVKPPPPPPRVCARCDSDNTKFCYYNNYSEFQPRYFCKNCRRYWTHGGALRNVPIGGSSRAKRTRINQPSVAQMVSVGIQPGSHKPFFNVQENNDFVGSFGASSSSFVAAVGNRFSSLSHIHGGMVTNVHPTQTFRPNHRLAFHNGSFEQDYYDVGSDNLLVNQQVGGYVDNHNGYHMNQVDQYNWNQSFNNAMNMNYNNASTSGRMHPSHLEKGGP

>AtDof4.4

MDNLNVFANEDNQVNGLKRPPPSRVCPRCDSDNTKFCFYNNYSESQPRYFCKNCRRYWTHGGALRNIPVGGSCRKPKRLKVDQSSISEMVSVENQPINHQSFRQTQENNEFVRSFDASSSATVTAVPNHFGYLSELHGVTNLLPIQSFRTMDCLDFGDESFQQGYYDVGSNDLIDNPLINQSIGGYVDNLTSYCINQVEPKLQPRYEHES

>AtDof4.5

MDNLNVFANEDNQVNDVKPPPPPPRVCARCDSDNTKFCYYNNYCEFQPRYFCKNCRRYWTHGGALRNIPIGGSSRAKRARVNQPSVARMVSVETQRGNNQPFSNVQENVHLVGSFGASSSSSVGAVGNLFGSLYDIHGGMVTNLHPTRTVRPNHRLAFHDGSFEQDYYDVGSDNLLVNQQVGGYGYHMNPVDQFKWNQSFNNTMNMNYNNDSTSGSSRGSDMNVNHDNKKIRYRNSVIMHPCHLEKDGP

>AtDof4.6

MDTAQWPQEIVVKPLEEIVTNTCPKPQPQPLQPQQPPSVGGERKARPEKDQAVNCPRCNSTNTKFCYYNNYSLTQPRYFCKGCRRYWTEGGSLRNIPVGGGSRKNKRSHSSSSDISNNHSDSTQPATKKHLSDHHHHLMSMSQQGLTGQNPKFLETTQQDLNLGFSPHGMIRTNFTDLIHNIGNNTNKSNNNNNPLIVSSCSAMATSSLDLIRNNSNNGNSSNSSFMGFPVHNQDPASGGFSMQDHYKPCNTNTTLLGFSLDHHHNNGFHGGFQGGEEGGEGGDDVNGRHLFPFEDLKLPVSSSSATINVDINEHQKRGSGSDAAATSGGYWTGMLSGGSWC

>AtDof4.7

MMTSSHQSNTTGFKPRRIKTTAKPPRQINNKEPSPATQPVLKCPRCDSVNTKFCYYNNYSLSQPRHYCKNCRRYWTRGGALRNVPIGGSTRNKNKPCSLQVISSPPLFSNGTSSASRELVRNHPSTAMMMMSSGGFSGYMFPLDPNFNLASSSIESLSSFNQDLHQKLQQQRLVTSMFLQDSLPVNEKTVMFQNVELIPPSTVTTDWVFDRFATGGGATSGNHEDNDDGEGNLGNWFHNANNNALL

>AtDof5.1

MVFSSFPTYPDHSSNWQQQHQPITTTVGFTGNNINQQFLPHHPLPPQQQQTPPQLHHNNGNGGVAVPGGPGGLIRPGSMAERARLANIPLPETALKCPRCDSTNTKFCYFNNYSLTQPRHFCKACRRYWTRGGALRSVPVGGGCRRNKRTKNSSGGGGGSTSSGNSKSQDSATSNDQYHHRAMANNQMGPPSSSSSLSSLLSSYNAGLIPGHDHNSNNNNILGLGSSLPPLKLMPPLDFTDNFTLQYGAVSAPSYHIGGGSSGGAAALLNGFDQWRFPATNQLPLGGLDPFDQQHQMEQQNPGYGLVTGSGQYRPKNIFHNLISSSSSASSAMVTATASQLASVKMEDSNNQLNLSRQLFGDEQQLWNIHGAAAASTAAATSSWSEVSNNFSSSSTSNI

>AtDof5.2

MADPAIKLFGKTIPLPELGVVDSSSSYTGFLTETQIPVRLSDSCTGDDDDEEMGDSGLGREEGDDVGDGGGESETDKKEEKDSECQEESLRNESNDVTTTTSGITEKTETTKAAKTNEESGGTACSQEGKLKKPDKILPCPRCNSMETKFCYYNNYNVNQPRHFCKKCQRYWTAGGTMRNVPVGAGRRKNKSPASHYNRHVSITSAEAMQKVARTDLQHPNGANLLTFGSDSVLCESMASGLNLVEKSLLKTQTVLQEPNEGLKITVPLNQTNEEAGTVSPLPKVPCFPGPPPTWPYAWNGVSWTILPFYPPPAYWSCPGVSPGAWNSFTWMPQPNSPSGSNPNSPTLGKHSRDENAAEPGTAFDETESLGREKSKPERCLWVPKTLRIDDPEEAAKSSIWETLGIKKDENADTFGAFRSSTKEKSSLSEGRLPGRRPELQANPAALSRSANFHESS

>AtDof5.3

MDHLLQHQDVFGNYNKAREAMGLSYSSNPTPLDNDQKKPSPATAVTRPQPPELALRCPRCDSTNTKFCYYNNYSLTQPRYFCKSCRRYWTKGGTLRNIPVGGGCRKNKRSTSSAARSLRTTPEPASHDGKVFSAAGFNGYSNNEHIDLSLAFALLNKQHPGSSSQLGFHSELGSSHQSDMEGMFGTSQQKENATYAFGNGSSGLGDPSRVLWGFPWQMNGESFGMMNIGGGGGHVDQIDSGREMWTNMNYINSGALM

>AtDof5.4

MQDIHDFSMNGVGGGGGGGGRFFGGGIGGGGGGDRRMRAHQNNILNHHQSLKCPRCNSLNTKFCYYNNYNLSQPRHFCKNCRRYWTKGGVLRNVPVGGGCRKAKRSKTKQVPSSSSADKPTTTQDDHHVEEKSSTGSHSSSESSSLTASNSTTVAAVSVTAAAEVASSVIPGFDMPNMKIYGNGIEWSTLLGQGSSAGGVFSEIGGFPAVSAIETTPFGFGGKFVNQDDHLKLEGETVQQQQFGDRTAQVEFQGRSSDPNMGFEPLDWGSGGGDQTLFDLTSTVDHAYWSQSQWTSSDQDQSGLYLP

>AtDof5.5

MLETKDPAIKLFGMKIPFPTVLEVADEEEEKNQNKTLTDQSEKDKTLKKPTKILPCPRCNSMETKFCYYNNYNVNQPRHFCKACQRYWTSGGTMRSVPIGAGRRKNKNNSPTSHYHHVTISETNGPVLSFSLGDDQKVSSNRFGNQKLVARIENNDERSNNNTSNGLNCFPGVSWPYTWNPAFYPVYPYWSMPVLSSPVSSSPTSTLGKHSRDEDETVKQKQRNGSVLVPKTLRIDDPNEAAKSSIWTTLGIKNEVMFNGFGSKKEVKLSNKEETETSLVLCANPAALSRSINFHEQM

>AtDof5.6

MGLTSLQVCMDSDWLQESESSGGSMLDSSTNSPSAADILAACSTRPQASAVAVAAAALMDGGRRLRPPHDHPQKCPRCESTHTKFCYYNNYSLSQPRYFCKTCRRYWTKGGTLRNIPVGGGCRKNKKPSSSNSSSSTSSGKKPSNIVTANTSDLMALAHSHQNYQHSPLGFSHFGGMMGSYSTPEHGNVGFLESKYGGLLSQSPRPIDFLDSKFDLMGVNNDNLVMVNHGSNGDHHHHHNHHMGLNHGVGLNNNNNNGGFNGISTGGNGNGGGLMDISTCQRLMLSNYDHHHYNHQEDHQRVATIMDVKPNPKLLSLDWQQDQCYSNGGGSGGAGKSDGGGYGNGGYINGLGSSWNGLMNGYGTSTKTNSLV

>AtDof5.7

MSSHTNLPSPKPVPKPDHRISGTSQTKKPPSSSVAQDQQNLKCPRCNSPNTKFCYYNNYSLSQPRHFCKSCRRYWTRGGALRNVPIGGGCRKTKKSIKPNSSMNTLPSSSSSQRFFSSIMEDSSKFFPPPTTMDFQLAGLSLNKMNDLQLLNNQEVLDLRPMMSSGRENTPVDVGSGLSLMGFGDFNNNHSPTGFTTAGASDGNLASSIETLSCLNQDLHWRLQQQRMAMLFGNSKEETVVVERPQPILYRNLEIVNSSSPSSPTKKGDNQTEWYFGNNSDNEGVISNNANTGGGGSEWNNGIQAWTDLNHYNALP

>AtDof5.8

MPSEFSESRRVPKIPHGQGGSVAIPTDQQEQLSCPRCESTNTKFCYYNNYNFSQPRHFCKSCRRYWTHGGTLRDIPVGGVSRKSSKRSRTYSSAATTSVVGSRNFPLQATPVLFPQSSSNGGITTAKGSASSFYGGFSSLINYNAAVSRNGPGGGFNGPDAFGLGLGHGSYYEDVRYGQGITVWPFSSGATDAATTTSHIAQIPATWQFEGQESKVGFVSGDYVA

>SbDof1

MEAPLHQFPVPPPLPQDALLRQQAVAARALMVAATAGNKAAGREQCPRCASRDTKFCYYNNYNTAQPRHFCRACRRYWTLGGSLRNVPVGGSTRKRPRPRPARHTRAMAAAAFGAAAMATPSTTTTASDGGSPFASPATFQGVGGGGGLLLSSLLLGSVSASSSASPLLALGAAPLLEGRLGFDLGFGDAALGGGGGGGAHAADLPHHHQLPLGGGPPLLPWPAATTRILEGDRAETTTVFPFPPAGAVWQELAATAPVVEAAGLHHGGAPHLLL

>SbDof2

MAECQGGGGGDFLIKLFGKTIPVPESGDAKDLQQSSSSSSSTEQDHQDAHALDQENPHPDSSDPSPQPEVVDAEDPKSSPETTHQKPGQGNGSGDAASQREKLKKPDKVLPCPRCNSMDTKFCYFNNYNVNQPRHFCKNCQRYWTAGGAMRNVPVGAGRRKNKNAVAASHFLHRVGAACGGGGDTLKTTNGTVLSFGGHGGGCVPPGPACLDLVEQLSHHLAAPVIRNAGNNPGPCSEGSSNCRDDNKTINDRSCVDEAAAANGDDGSVQHPASMNNGGATVWPPPYSCAPSPAAYFSSGIAIPIYPAAPGYWGCMVPGAWSLPWPVQQPPSSQSQGPAAGLSSSTSPTTTSAPSVSSSGAADSHTLGLGKHPRDREEGDDGRNAKVWAPKTIRIDDVDEVARSSIWSLIGIKGDKAKQQDDDAAGGHKQKQLVGMVFEPKREATKKPAAMMTSSPLLHANPVALTRSVAFQEGS

>SbDof3

MAERARLARMPQPEPALKCPRCESTNTKFCYYNNYSLSQPRHFCKTCRRYWTRGGSLRNVPVGGGCRRNKRSSKSSSAAAAGSSSSSKTSSSGRLLGGPSATPSTTPGVTGAIITPGLSSFSHHHLPFLGSMHPPGPNLGLAFSAGLPLLGMQHLDTVDQFPVASGGGTTIGASLEQWRVQQQQQRQFPFMTGGILDLPQPPTYQLGLEANRGGGGGSAAAAFTLGQPTTTSATATTGRQEGSPKKMEDSKGQDMSLQRQYMAALRHGSGAHGGWDGNAAGGSGSDGGGTGSAGSTWPMNIIPGFHSSSTTGGNGGGSL

>SbDof4

MAAVVAASGGGSTPGAGGGPTAGGSAIRPGSMTERARLAKIPQPEPGLKCPRCESTNTKFCYFNNYSLSQPRHFCKTCRRYWTRGGALRNVPVGGGCRRNKRTKSSKSNSSSAASASGAGGTSSSTSSTATGGSSAGGASGAGIMPSHQGHGHLPFLASLHHPLAGGDHYSTGASRLGFPGLSSLDPVDYHQFGASAGAGGAALGLEQWRLPQIQHQFPFLSGRPDAVPPTMSGIYPFDVEGHGGDGTGFAGHMLGASKVPGSAGLITQLASVKMEDNPASAAMANSSPREFLGLPGNLQFWGGGSNGGANGNNGGGAGNAGGGGSGGGGGGGGGTVAPGSSWVDLSGFNSSSSGNVL

>SbDof5

MASATTAATGDDAVGTRKGGTGGATAGGGTAPQTQQQTPPPPEQGLRCPRCDSPNTKFCYYNNYSLSQPRHFCKTCRRYWTKGGALRNVPVGGGCRKNKRSRSAAAVAVAAAAAASRLSLNLPPAEAAAADQQQAAARLGFLGAAHHHPAVASSPIGGAGAPAADYHHQQAGMMALQPRLHGPGAVVGQYVPFGDWPSSGDVSSGGGCHAVNGGAAAAVSMSSSIASSIESLSFINQDLHWKLQQQRLATMFLGTPTTSSGAAAGAHVDGAAAAAGAAAPHVVGGTFLHMAAGPPPPHGMVEATTMAPPAATSWFMDSSCYVLPSPTTAHHANTAAAAVVATSNCNVVNSSGGGDDDNATSNNNNCGPGSAIPSWGDMSTFAMLP

>SbDof6

MAGAGGATAVQQPAAGAPAAAGGAARSGVGAGAAGAPVADPRAEALRCPRCDSANTKFCYYNNYSLSQPRHFCKACKRYWTRGGTLRNVPVGGGCRKNKRSRSSGGAGGRNGSSSSASAAAAAVTSSSAASTLSLPPPTGSLPSLTSALGLPGGASLASLLLGTAGSGGDHLGLFQAAMQSVVSSEATAYEMQQQQSQVDHLLGLGYGGATGAGAQIHLKPWMQEAAGAGGIMDSFYAPLLSSSLVPGLEELHVKAEVAGAGDHQQKPAPGDQQSASWELPTPSSSNVDANVIASDALMAAAAASMNPAVSSSTSTAPTTVPSSFMYWGNGGIGGAAAAWPDLANCGSSIATLF

>SbDof7

MQEHGRRPVTPFAGVDLRRPKGYPAPAAVAAAKEAAPARAPVVGDPCPRCGARDTKFCYYNNYNTSQPRHFCKSCRRYWTKGGSLRNVPVGGGTRKSSSSSTSSPSATTSASPGGAAPKNTKRSKNSKRRRVAPAPDPAAPGTDAPTVTATATADVANTAPSTEAAAATVAASEKPVTMTEEEPAAAVVVATETKPPAAPGLGLADAGSGGGGKELLPDPSHFEWPSGCDLGSYWGTSVFADTDPALFLNLP

>SbDof8

MRVTGEKPCTHQELDVDQTNSSSSFNNSSECENLAPSNDEISGSESNLEIAKTEGDVPSGEKVLKKPDKILPCPRCNSMDTKFCYYNNYNIKQPRHFCKSCQRYWTAGGSMRNIPVGAGRRKSKSSSANCRSILIPGSSVATPGGEASLFPLSINGNQAAVSFGPDSPLCNSMASVLKIGGEQIKSSNPASAAQPRNGENQMCPPCTTSSDGPRNESQKETANAHQNGIIGQSNGVTSVHPIPFFPGPPFVYPWSPAWNGIPTMAAAVCPAPAEAANSSENCTTSSNVQWNVPPIVPVLPPGFCGPIPVPVMPPSVWPFITPWPNGAWNAPWLGPSSTVPSSSPTSSSTCSESGSPVLGKHSRDSKPQGDEKAERCLWIPKTLRIDDPVEAAKSSIWTTLGIEPGDRGMFRPFQSKPERQEQISGAARALQANPAALSRSRSFQETT

>SbDof9

MAGQVMEAQAARLQPPTMLAPPFAPLPHSTTCKHDVHHHHLTTTTTTATMAATSGTTTNNVVTRTGGAAAADMAAYLQQLQDAAEAAAKSSGGTGGAARGEQCPRCASHDTKFCYYNNYNTSQPRHFCRACRRYWTLGGSLRNVPIGGSTRKRPRLAHHQHQQHARRAPAAAAAAHVFGLGLGLGGAAPPPMMMMPPLPCSSSSSSLQGQGQGQGQGGGGLLGSLFALGAAGAPPLLEGRGAGSSSSFDFDLGLGLPTAGPLHLGEAAAAVQMQGLGLRGGGGNGNAAGSSSSFLWPAGLLADNDDSVDTWTKMPPGAGAGSMWPDFFSSPPAAAAPQTGGMMLHGGAHLM

>SbDof10

MDQLKSVNDVNAASLPLLLHPVISNPSPTSSSSTSSRSSAQQQQRSTSATSSPQGQGPQQQGQGQGAEQTPLRCPRCNSSNTKFCYYNNYNLTQPRHFCKTCRRYWTKGGALRNVPIGGGCRKPRPMPTPVAKPVISSCKAVGGGVPSLGLGVGLGMGMGAGPGPWASSQQAAAAQLMALLNSARGVHQGGGNMHRLLGLDAMAHLPLHVLPGAGNNNAGGTAPSLWPQSAPRAIPTPPHMDSQLGMGPLGQHDVLSSLGLKLPPPSSSPAAASYYSDQLHAVVSSAAGRGGHEYEAAASGAMSLPCTTALTSLPPAASSVSAALTSCATVGLDLPPVSVPASEMQYWAAGPAAMSVAWPDLPTPNGAFP

>SbDof11

MLSSHHEAMLPYAPGPPPSLLVDRRYKQGGEAAPNCPRCDSPNTKFCYYNNYSLSQPRYFCKGCRRYWTKGGSLRNVPVGGGCRKNRRGKSSLRSAADAAIASGGGRDAAFGHHRFPGPVRPDLVLEGMVGNPSNPAGQAMPGGVPAATDGSTIDLAMLYAKFLNHPPAEEGVNAVTPESAGQVVDEAFDTFSASSDLSPGVLAPLQFDPCHDGFGEWSAGGPVSSTGPSSTASTTAATTMLCADVSVQAAFGELNFAMDQSCFDSLGLPTDDVVGNLSSSWCSIVPGLSTLEDTKYDSLDSFPDDALSLHEDMISGTDHDWSVDCQGLEALYMP

>SbDof12

MAPAAGGFKLFGKVITRTQCAAETAPPAVPTQEQEPAAAASRSTAAFARERDDPDERDQPMVKREAAAAAASDHDFVVVADKQQQQHSAAAGGPAPASESDDSKGQQQHPRPRQHHQQQHQDTVEARAAAAASSAPPLPCPRCRSRNTKFCYFNNYNVNQPRHFCKDCHRYWTAGGALRNVPVGAGRRKNRPLGPVGAVPVPVPVPVPVPAHLHPAAAAGFVLGFPGQHPSSPTTSPSTAVYAERWPVCPDRRF

>SbDof13

MASHLPDADAAGFKLFGKVIQPPDAHHRAADEGGAPPQLPPPTTALPPPPPPPPSPPLPPQPPLPLQQQATGATGGTSGGGGEPLPCPRCGSRETKFCYFNNYNVRQPRHLCRACRRYWTAGGALRRVASASPGRRSPRPTSARSVAAAAAAAAAASSAAAAAAEEVGGER

>SbDof14

MAAPGGGLDQDRGRGRGQDAAIKLFGRSIPVLHSSVVAAAASEVSTKLANDVRSNDGMSCLPNMPLIVKASPFPSKNNMKNGLQAISSQHGKMEADSKSEEVKNSLQAIISQPGKTDDSKSEETKTESGGSGQEKVLKKPDKILPCPRCHSMETKFCYFNNYNVNQPRHFCRNCKRYWTAGGTMRNVPIGSGRRRNKDPSHHHHVTKPCDHIVTANGDVSDATQRQSLAVKPSVLQGSGKQNETACKSVSPVLNIKEQNNADLISLVSGDNKEEKSCASSVVSGSSENWMPENTVKKEEDSTSAYGNGVKEPDPNTQSHHAGPISVFSGNPAAVMVTNQSSADGIHGPGNGTVSPLSLPPPPMVPTPGICAPAVPFPLVPAFVSCIPGWPSAVWGAAWPGSSGPTLLSLPPNSLAFSGSNSRVLGKHTRVANLQEEQKAEKKFWVPKALRIDNPEEAAKSSIWASLGIKPDERIIFKSFQSKDLKNSETKTPESLQANPAAFSRSQTFQERT

>SbDof15

MSDQKDPGIKLFGRVIPLEPEPAPGTTEAEDPPPSHDQPPDELQPRAPELAAAADEDQHNEKEEKPASEMVNMPQEKDKEIKVDTPQVEKDNEMKVDAPQKEHDDEMKIDAQQEKKDEQMEVNGSPMHENIEPANLPPSEHKKEDEDLMNSTEDKAASDPKGENEKTSNEESGQDKALKKPDKILPCPRCNSMDTKFCYYNNYNVNQPRHFCKNCQRYWTAGGTMRNVPVGAGRRKSKNSSLHYRQLLMAPDCMLGSRVDISKSVLPEALVSPPAPIQPTSRNETVLKFGPEVPLCESMVSALNIDEQNVNNSGSAPRGENREDNPGPGTSYNGVPENMVHVDKNGAPVHCNGVAPVPQYYLGTPFMYPWNVGWNNVPVMAPASRFPSPAFPYPLVPPALWGCLSGWPVTTWNIPWIRANGCVSPSSSSNSSCSGNGSPTLGKHSRDSNPMKEEKREKSLWVPKTLRIDDPDEAAKSSIWATLGIKPGDPGTFKPFQSKVESKGQRSDAAQVLQANPAALSRSQTFQESS

>SbDof16

MEEMLMAGNANPNQNPNPPPPAPSAPGAQRAGAPAAGAAAAPSAGATGGPAGAGTERRARPQKEKALNCPRCNSTNTKFCYYNNYSLQQPRYFCKTCRRYWTEGGSLRNVPVGGGSRKNKRSSSAVSSAAAASTSAAMSGTVSVGLPAKNPKLMHEGAHDLNLAFPHHNGRALQPPEFPAFPSLESSSVCNPGAAGMVGNGAAGRGMGALSAMELLRSTGCYVPLQHVQLGMPAEYAAAGFALGEFRMPPPPQSHSVLGFSLDTHGTGGVGGAGGYSAGLQDSAAGRLLFPFEDLKPAVSAAAGGGGASNGADHHQYEHSKDQAAGDGGSGPSGVTGGHETPGFWSNSLIGNGSSNGGGGPW

>SbDof17

DPIKSTREPLYTYPNREKLNETLGVLIKDEAKHKGIVPEEPGMGSSSPSAELIACPRPMHAVAAAAAADRRLRPQHDQPLKCPRCESTHTKFCYYNNYSLSQPRYFCKTCRRYWTKGGSLRNVPVGGGCRKNKRASAKKPPAPPMQPRHMAETGLHLSFSGMPQLPPPSADPLCSLGLLDWKYDPILTGSGGGAAAGSLDGASSEAHFAGAGMLGIPGGGGSGGECHALSALRYAAGLGEHLQLQGLPFGGRAEHDGMEVKPPATERLLSLDWYSETSRAPESAISSLGALGLWSGMLGGAHQHHGSSAAI

>SbDof18

MQEFQSIPGLAGRLFGGAGAGDLRRAQAHAQQGPGARCGGVSPAAPPEVVKCPRCESTNTKFCYYNNYNLSQPRHFCKSCRRYWTKGGVLRNVPVGGGCRKTKRASSSSSATSSVPSTPTSGSGDAAANKNPRRASASSPRSTNSGSASPTAAAATTPTPAPTTPATPSSNSSVAVFTTSSHHHSSPFSTIDVVAPPAPIFADQAAARASLFAPPPPPPLPVFTFAAQPKQEEAPTTTSELQLVAGLSAAAPSSSSVVSEDMAPFASLDAAGIFELGDAASAAAYWNAGSCWTDVVQDPSMYLP

>SbDof19

EAIVSSPIIKEEARSPKQAQVTQQASGSGERKPRPQLAEALRCPRCNSNNTKFCYYNNYSTSQPRYFCKGCRRYWTHGGALRNVPVGGGCRKNKRTSGSISASGTSSSSSAAYAPLSPSTNTSSSKMSINTQLMMVPNMMMSTSSMTGLFPNVLPTLMSATEGGEFNFTMDNQHASLPFTPMSLSNQASVPVLAAGESGTMPSFLEMLRKGLLHGSSSYDTGLAMSDGNNGMDMSFPLPAYGAMHGHGLSGSTTNDARQLVGTQQGVNTGGGFVGSTGVQEEEEEGDNKAMVKSNKNNNGGSLLDRYWIKPNNNNNKRQQG

>SbDof20

MEEMLMAANAGAANPSQGSNNPNPPAPAPGGALRGGGAPAAPLAGAGSTERRARPQKEKALNCPRCNSTNTKFCYYNNYSLQQPRYFCKTCRRYWTEGGSLRNVPVGGGSRKNKRSSSSASASASTSASVTSSSMASAEGAAASKNPKLAHEGAHDLNLAFPHHGGLHAPEFAAFPSLESSNVCNPGGGMTSNGRGGGAGPAVGALSAMELLRSSGCYMPLQMPMQMQGDYTAAEFALGDFRTPPPPPSQSVLGFSLDAHGPGSGAAAAGYGSSAGLQGVTENAGRLLFPFEDLKPPVSSGGGGVAGGATGGAGDGNSNHNQFDHNKEQDGGGGPGAGHDTPGFWSGMIGGSGASW

>SbDof21

MAPAASILSATAAAAGASKRPADSDAELSLDSSALQQQGDEAVRKGRQTRQQQQLECPRCRSTNTKFCYYNNYSTAQPRHFCRACRRYWTHGGTLRDVPVGGASRRSSGGGGKRRRVSAEPSSAASSSPPPLPAASLADACLPDLTSAFPFLSDGSFFPQFDVGSGVALAPAAFSSSWQSVVPDFYDGLAPWDDGAAAGTAAGGFVGAWGDIAGLDLSWTPPGN

>SbDof22

MDGVSTSPSSVTATATIREKPKPAPAATAPASLYLAVLRPSGSPSPSLSLYATGPRASQPEPGTTAPSRMQEVSVEPGRRPAQQPHHQFAGVDLRRPKGYATAPVQEATPAVKVAEGGDPCPRCASRDTKFCYYNNYNTSQPRHFCKGCRRYWTKGGTLRNVPVGGGTRKKPSSSSYAAAAAAAADADNKPPKKKPASKKRRVEAPVPEPAAAADASGVTDTVAAADSAKTTTTTTTTTTDGASEITTETEAAVAVAVPVAEEDSESLAHLLLQPGAEEAVSLGLGLSDFPSAAGKAVLDDDSFVWPAAFDLGTCWASAGFADTDPASLFLNLP

>SbDof23

MGEGRAGDGLIKLFGKTIPVPETPAAGDAAKDIQQSGSSGTTDLKGQENTLQDSTGSPPQQEVADTEDSSAAKNSSADKQQGEAANQKEKLKKPDKILPCPRCNSMDTKFCYYNNYNINQPRHFCKNCQRYWTAGGAMRNVPVGAGRRKSKSASAASHFLQRVRAALPMDPLCTAAKTNGTVLSFGSDMSSLDLTEQMKHLKEKLIPIAGIKNGDERSVGSCTEGPAKAEDSNQKENVTAEKSAKLVQHPCMNGVAMWPFSCAPPPACYTPGSIAIPFYPAPAAYWGCMVPGAWNAPWPPQSPSETGSTLSTASPASTKSNCFTPGKRPRDCNEEGDTKGNGKVWVPKTIRIDDVDEVARSSILSLIGINGDKAGKDGKGCKLARVFEQKEEARTATHSVINGLPFLQGNPAALSRSLTFQEGS

>SbDof24

MVSSPNIKEEARSPKQAEATQQPSGSGERKPRPQLAEALRCPRCNSNNTKFCYYNNYNTMQPRYFCKGCRRYWTHGGTLRNVPVGGGCRKNKRASRSVSGSGSSSSSAAYAPLSPDTNTSSSKMSINTQPMMVPNMMMPTPTMTGLFPNVLPTLMSTGGGSHFNFTMDNQHASMPFTPMPLSNQASVPMLAAGGSGTMPSFLEMLRRGLLHGSSSYDAGLVMSGGNNEMDMSFPLPAYGAMHGHGLSGSTTDDARQLVGTQQGMNTDSGFAGSTRVQEEEEEKGDNKAMVKSSNNNNAQQQQQQQQQQQQQQQQQQQQGAAGVVCQAVEVLLLQTI

>SbDof25

MKLSSSSHHLLLESSNLTLSPHHRNTAVVTRMLSSHCENMLPYAPGRRAAVLLDHRRYRPNVVEVAPSCPRCDSPNTKFCYYNNYSLSQPRYFCKGCRRYWTKGGSLRNVPVGGGCRKNRRGKPVVRAVAVDAAVAAASGGASALANRSSSSSPATLRPDLLLEGMIGSPVGLCQPTDDDAAEIPAVVAPEGSTIDLALLYAKFLNHQPAAAAEPCAAAVVPESLDTLSGSSTSGDVSPVVVPPRDQHQPFTTQDHGGFGELSATTASAEPSAAPPQRPADDACACAAEALIGALSVDPRCYDSLGLPPDGGDLVLPSTWHLGTKYEPFDPLPEDAMSLQDGFAGDEDVWSSALACQGLEAALCRP

>SbDof26

MGGPLPDGGGGGGGGAGGQVVGGPAKPMSMAERARLARIPLPEPGLKCPRCDSTNTKFCYFNNYSLSQPRHFCRACRRYWTRGGALRNVPVGGGYRRHAKRAKPKQQQAAGAGAGGGSAGTGNTATANAALQHAPAGSTASSAAACTATTTNALPGGMLGGGGLSMLPPLLRLADFDAMSLGSTFSGISSMGKPGSIDAYSHSVGGGGAPAGLEQWRVQQMQSFPFLHAMDQGPLGPPLAMAMAAPGMFQLGLDTTSSDNGHGRGGGGGEDGSTGGELHVMHQQAATKRESYPAPRGMYGDHHHHLAAAGGYTSAYSTNAAT

>SbDof27

MEAGQVPDGRALMAAVTTTGGGGREPEGLPCPRCESVNTKFCYYNNYNLSQPRYFCKTCRRYWTRGGALRNVPVGGNTRKATPATGRRKRSTPAPVNVTVPAPATASPPPPPALHGGSLLRPYGGGGGSGLLSFAAPALASPLAAADPDRRLLDFGGSFTSLIAPGVADVGVHFSAGFLMGGLAPAALPRAPGSVAALPPPPPQQQPTVSQALPEGMVWSMGWPDLSI

>SbDof28

MQMQQQPPLQCLLGGGGGGSDHHHLMPPPSGLAPLPGGPADTAASAPAGGGSSTSMQAAAGAGTAAAQPRPVVSMAERARLARVPLPEPGTLRCPRCDSTNTKFCYFNNYSLSQPRHFCKACRRYWTRGGALRNVPVGGGCRRNTKRSSKKSSRGGGGAGATAATSSSSTTSTSTTATTTTATTTSAAMAAAEAIASMQAQLPHLGLPPAAAAAALEASLEGYHHYLPLQMQPQFLQQAGLHGYHFADDGTGVLADGFPRGVVASGLLAQLAAVKMEEHSSNGGGAVAAHHEQSYWPGSTGGGSGWPAEFLSGFSSSSSGNVL
