# Supplementary material for: Allele specific expression of Dof genes responding to hormones and abiotic stresses in sugarcane
Source: PLoS One. 2020 Jan 16;15(1):e0227716. doi: 10.1371/journal.pone.0227716 (PMC6964845; doi:10.1371/journal.pone.0227716)
Supplement: S4 Table — (DOCX) [file pone.0227716.s004.docx]

Total numbers of cis-elements on promoters of *SsDof* genes.

|  | Function | Elements | Number |
| --- | --- | --- | --- |
| Growth and development | light responsive | G-box | 557 |
|  |  | Sp1 | 181 |
|  |  | Box 4 | 144 |
|  |  | GATA-motif | 72 |
|  |  | TCCC-motif | 78 |
|  |  | TCT-motif | 67 |
|  |  | GT1-motif | 74 |
|  |  | GA-motif | 24 |
|  |  | I-box | 71 |
|  |  | ATC-motif | 10 |
|  |  | ATCT-motif | 15 |
|  |  | AE-box | 29 |
|  |  | Box II | 24 |
|  |  | GTGGC-motif | 22 |
|  |  | ACE | 35 |
|  |  | LAMP-element | 10 |
|  |  | 3-AF1 binding site | 4 |
|  |  | AT1-motif | 2 |
|  |  | chs-CMA1a | 4 |
|  |  | chs-CMA2a | 5 |
|  |  | chs-Unit 1 m1 | 2 |
|  |  | CAG-motif | 3 |
|  |  | L-box | 1 |
|  | MYB binding site | MBS | 100 |
|  |  | CCAAT-box | 83 |
|  |  | MRE | 34 |
|  | endosperm expression | GCN4_motif | 10 |
|  | cell cycle regulation | MSA-like | 6 |
|  | zein metabolism regulation | O2-site | 62 |
|  | seed-specific regulation | RY-element | 68 |
|  | meristem expression | CAT-box | 154 |
|  | circadian control | circadian | 30 |

Additional file 4 (continued).

|  | Function | Elements | Number |
| --- | --- | --- | --- |
| Phytohormone response | abscisic acid responsive | ABRE | 532 |
|  | auxin responsiveness | TGA-element | 79 |
|  |  | AuxRR-core | 38 |
|  |  | TGA-box | 1 |
|  | gibberellin-responsive | P-box | 51 |
|  |  | TATC-box | 12 |
|  |  | GARE-motif | 37 |
|  | MeJA-responsive | CGTCA-motif | 334 |
|  |  | TGACG-motif | 334 |
|  | salicylic acid responsiveness | TCA-element | 81 |
| Stress response | anaerobic induction | ARE | 194 |
|  | anoxic specific inducibility | GC-motif | 122 |
|  | defense and stress responsive | TC-rich repeats | 49 |
|  | low-temperature responsive | LTR | 76 |
