# Supplementary material for: Allele specific expression of Dof genes responding to hormones and abiotic stresses in sugarcane
Source: PLoS One. 2020 Jan 16;15(1):e0227716. doi: 10.1371/journal.pone.0227716 (PMC6964845; doi:10.1371/journal.pone.0227716)
Supplement: S6 Table — (DOCX) [file pone.0227716.s006.docx]

Sequences of the primers used in this study.

| Gene name | Forward primer (5’-3’) | Reverse primer (5’-3’) |  |
| --- | --- | --- | --- |
| *SsDof3* | GTCGTCGTCCTTCGACTTTG | CCCGAGGCCTTGCATTT |  |
| *SsDof4* | CGACCATGCATCATGCTAAC | ACTCCCGCAGTTGTTGTT |  |
| *SsDof5* | GCTCGATCCATCGCCTATATAAC | CAGTACCTGACGAAGCAGAAG |  |
| *SsDof10* | GGGATCATGGACAGCTTCTAC | CTGGCTTCTGCTGGTGAT |  |
| *SsDof13* | AACATGGTTCCCGTCGATAAG | TTCCAAGGGTACATGAAAGGG |  |
| *SsDof17* | GTTAGAGGACGCCAAGTATGAC | GCATGTAGAGAGCCTCCAATC |  |
| *SsDof18* | GTGGCCAGGAGTTCTATCTTG | CGTTGATGACTGGGTGAGTT |  |
| *SsDof20* | CAGTACGAGCACAGCAAAGA | GCCATTCCCGATCAAGCTAT |  |
| *SsDof22* | GCGGTGTTCTTCGTCTTCT | GTACATATTCTCCTGCTCCATCTC |  |
| *SsDof23* | TCGGTGGTCCCGGATTT | AGTCCAGCTGAGGTCGAG |  |
| *SsDof24* | GGACGACTCGTTCGTGTG | ACGGGAGGTTGAGGAAGA |  |
| *SsDof28* | AGTGCTGTGGCTGGAATAG | GGTGGAGATTCCCTTAAGTACC |  |
| *GAPDH* | CACGGCCACTGGAAGCA | TCCTCAGGGTTCCTGATGCC |  |
